# On the Increased and Decreased Structural Connectivity of the Demented Human Brain

**Published:** 2026-07-06

**Authors:** Dániel Hegedűs, Márton Barnabás Móra, Bálint Varga, Vince Grolmusz

**Affiliations:** a PIT Bioinformatics Group, ELTE Eötvös Loránd University, H-1117 Budapest, Hungary; b Uratim Ltd., H-1118 Budapest, Hungary

## Abstract

With the enormous advances in cerebral imaging techniques, a large amount of data is available for studying the aging and demented brain. In this contribution, we apply the OASIS-3 dataset for identifying small areas of the human gray matter, which have higher- or lower structural connectivity in dementia and aging. As anticipated, we found that finer structures of the hippocampus and the temporal lobe show decreased connectivity in dementia. More surprisingly, the precuneus, the cuneus, and finer structures in the insula show higher connectivity in dementia than in the healthy state.

## Introduction

With the higher human life expectancy, diseases that are related to older age cause much more concern worldwide than several decades ago. Age-related anatomical changes have been well-documented in various organs [1], including reductions in the weight and volume of muscles, lungs, kidneys, and the liver. Bone mass decreases, skin becomes thinner and less elastic, and cartilage degeneration leads to painful joint movement. Additionally, arterial walls thicken and harden, leading to cardiovascular aging. Understanding these age-related physiological changes has paved the way for numerous treatments, novel medications, and surgical interventions, all aimed at promoting healthier aging and prolonging independent living.

The human brain and the brain connections also change in the aging process. The functional changes are frequently connected to anatomical aging [2]. Several studies have reported age-associated reductions in cortical volumes [3] or sub-cortical gray matter volumes [4], and changes in anatomical brain connections [5, 6, 7, 8].

One of the most important age-related changes in brain function is dementia. Dementia is uncommon before age 60, but its prevalence doubles approximately every five years thereafter [9, 10]. It affects around 40% of individuals over 90 and up to 20% of those between 75 and 84 [11, 12]. According to recent World Health Organization (WHO) estimates [11], 55 million people globally have dementia, with 10 million new cases diagnosed annually. The leading cause of dementia is Alzheimer’s disease (AD), which initially manifests as forgetfulness, disorientation, and impairments in concentration, calculation, language, and judgment. As the disease progresses, some patients experience severe behavioral disturbances and psychosis. In its final stages, individuals with AD lose the ability to care for themselves and become bedridden [11, 12].

The economic impact of dementia is also staggering, with an estimated yearly cost of 1.3·10^12^ $ US worldwide (estimation made for year 2019, [13]), including direct medical costs, direct social sector costs, and informal social costs.

In the present contribution, we made comparisons in global and local properties of the anatomical brain connections of demented and healthy subjects on a large publicly available dataset of https://braingraph.org [14].

### Braingraphs

Connectomes, also known as braingraphs, have become important tools in the research on the human brain. Two primary approaches exist for their construction: functional connectomes derived from functional MRI (fMRI) and structural or anatomical connectomes based on diffusion MRI. In this work, we focus exclusively on the latter.

Diffusion MRI enables the characterization of macroscopic water molecule diffusion within neuronal structures. In white matter, water molecules predominantly diffuse along the direction of neuronal axons, allowing for the identification of axonal pathways connecting different regions of gray matter within the cortex and subcortex.

By labeling these gray matter regions with their anatomical names, a connectome or braingraph can be constructed as follows: the nodes represent anatomically labeled gray matter areas, commonly referred to as Regions of Interest (ROIs), and edges are established between two nodes if a diffusion MRI-based tractography workflow [15] identifies an axonal bundle linking them.

This process effectively transforms MRI imaging data into a discrete graph structure, enabling the application of the extensive and well-developed field of mathematics, called graph theory. Originally introduced by Leonhard Euler in 1741 [16], graph theory has matured significantly over the 20th and 21st centuries, thanks to contributions from numerous mathematicians [17].

Our research group has computed and published multiple sets of undirected and directed braingraphs, each containing up to 1015 vertices per brain [18, 19, 20, 21, 22], derived from various public data releases of the Human Connectome Project [23]. These datasets, available at https://braingraph.org, have been widely used in structural analyses of the young and healthy human brain [24, 25, 22, 26, 27, 28, 29, 30, 31, 32, 33].

As the public releases of the Human Connectome Project provide the MRI data for young and healthy individuals, those public datasets do not facilitate the study of the aging brain. Here we also examine braingraphs from Section A at the site https://braingraph.org/cms/download-pit-group-connectomes/ [14], for studying the aging healthy and demented human brain [14]. The data source of those graphs is the public release of the OASIS-3 dataset [34], which contains MRI and PET records of 1098 subjects aged between 42 and 95 years. The data recordings entail a period of 15 years on 3T Siemens MR scanners at the Washington University Knight Alzheimer Disease Research Center [34].

In the present contribution, we evaluate several relevant graph-theoretical differences between the graphs of the healthy and demented brains.

### Braingraphs and dementia

Today dementia is most often diagnosed with the help of clinical syndromes and cognitive tests [35]. Medical imaging, mostly MRI, is applied in auxiliary roles to rule out brain tumors or hematomas, and evaluating the possible vascular damage, microbleeds or ischemia, next to the atrophies of cerebral regions [35].

While the graph-theoretical changes are not yet applied to diagnose dementia in the clinical practice today, numerous works deal with those changes in the literature, for example [36, 37, 38, 39, 40, 41, 42, 43, 44]. Most of these works analyze the connections between braingraphs and dementias, in particular, Alzheimer’s disease, with tools and terms originated from network science. In the present work, we compare graph-theoretical properties between healthy and demented braingraphs, which are robust against measurement and processing errors.

## Methods

In the present work we analyze the braingraphs published in the Section A at the site https://braingraph.org/cms/download-pit-group-connectomes/ [14], computed by our research group.

### Braingraph construction from the OASIS-3 dataset

The data source is the public release of OASIS-3 dataset [34]. The resource contains MRI and PET imaging recordings together with rich clinical data, from 1098 subjects between age 42 and 95 years, over a 15-year time span. Diffusion MRI data of 1472 sessions were recorded using Siemens 3T scanners of two models: TIM Trio 3T, and BioGraph mMR PET-MR 3T at the Washington University Knight Alzheimer Disease Research Center [34].

### Computational Workflow

The computation of braingraphs involved the identification of anatomically labeled gray matter areas (parcellation) and the computation of axonal fiber tracts (or streamlines) which connect those gray matter areas (tractography). The resulting braingraph has a vertex set corresponding to the anatomically labeled gray matter areas, and two such nodes are connected by an edge if the tractography step finds at least one axonal fiber that connects them. The gray matter areas were labeled by the FreeSurfer segmentation tool.

For the computation we have applied the Connectome Mapper Tool Kit v.3.1., abbreviated CMP3.1 [45, 46], with probabilistic tractography. CMP3.1 was applied with diffusion spectrum imaging (DSI) modeling, with one million streamlines. The most relevant MRtrix3 tractography parameters were mrtrix_tracking_config.min_length= 5.0, mrtrix_tracking_config.max_length=500.0, mrtrix_tracking_config.angle=45.0, mrtrix_tracking_config.cutoff_value=0.05.

From each MRI data set we have prepared 5 graphs with different resolutions with 124, 170, 272, 502 and 1058 vertices with the FreeSurfer segmentation tool, according to Lausanne2018 brain parcellations [45, 46].

We were able to process the data of 696 subjects from OASIS-3; the remaining subjects have one or more missing or erroneous files, which prevented their processing with the CMP3.1 workflow. More than one MRI were processed from numerous subjects, therefore, we have computed 975 graphs from the diffusion MRI data: one dataset from 482 subjects, 2 datasets from 156 subjects, 3 datasets from 52 subjects, 4 datasets from five subjects, 5 datasets from 1 subject.

In the braingraphs deposited at the https://braingraph.org site, the nodes and the edges have several attributes, detailed in the Appendix. In the present work, we make use the anatomical labels of the vertices, and the edge-weights describing fiber numbers, that is, the number of axonal tracts identified between the endpoint of the edge by the tractography phase of the processing. This quantity corresponds to the width of the connection, which also can be represented as a strength or the importance of a connection [47].

### Assigning “demented” and “healthy” labels to the graphs

The Oasis-3 dataset [34] contains psychiatric diagnosis for each imaging session for each subject. Since some of the subjects were evaluated several times, they have several associated braingraphs computed, corresponding to multiple recordings. We have assigned the “healthy” and “demented” labels to the braingraphs in the following way:

If any of the cognitive diagnoses was “demented” of the person, then all of his/her graphs were labeled “demented”. Otherwise, the graph was labeled “healthy”.

We note that this way the “demented” status corresponds also to pre-dementia, that is, persons, who will develop full dementia in several years interval. Our results, consequently, correspond to anatomical changes in dementia and also in pre-dementia.

This way we have assigned the to 351 graphs the “demented” and 624 graphs the “healthy” labels.

### Some graph-theoretical notations

In this work we examine the weighted degree of the braingraphs. The weights are assigned to the edges, and correspond to the fiber number.

### Weighted degree

Suppose that we are given a graph G with edge-set E and vertex-set V. A widely known and applied quantity is the *degree* of a vertex v, which is defined as the number of edges, adjacent to v; this non-negative integer is denoted by d(v). If we have a weight-function w on the edges, then we can generalize the vertex degree to *weighted degree $d_{w}(v)$*, as the sum of the weights on the edges, adjacent to vertex v:

$$d_{w}(v)=\sum_{\{v,w\}\in E}w(\{v,w\}).$$

In a sense, if a vertex has large degree, then it has some “importance”, since it is connected to many other vertices. If a vertex has a large weighted degree with a non-negative weight function w, then it also has some “importance”, since it is connected to other vertices with high-weighted edges.

We will use the weighted degree for braingraphs with the fiber number weight function, denoted by fn. In this setting, the weighted degree $d_{fn}(v)$ of a vertex v is the total number of the axonal tracts (i.e., fibers), which connect v to its neighbors.

## Results and Discussion

First we list the areas in the brain, which correspond to graph vertices, whose weighted degrees have changed significantly in dementia. As it is expected from the literature, the hippocampus, and several other gray matter areas found to be connected with significantly less weights in dementia, than in healthy subjects. However, we have found (also in line with some, but much fewer references in the literature) that some connections are stronger in dementia than in healthy persons.

The word “significantly” means that Student’s t-tests gives p-values less than 0.01 for all rows in the tables. In the Appendix we list the weighted degree changes for all vertices, while here we list only the vertices with largest changes in Tables 1 and 2.

We need to emphasize that in these tables we quantitatively, on a large dataset (351 demented, 621 healthy graphs) computed the weighted degree changes for all cortical and sub-cortical gray matter areas. Naturally, several nodes with the largest changes were already addressed in the literature, but most works focused to one or two such areas, and very seldom presented quantitative results on a large dataset.

Several areas of the hippocampus can be found in Table 1 with the lowest D/H ratio, meaning that for that graph vertices, and for those corresponding areas we found the largest loss in fiber numbers, represented by edge weights. It is well-known, that hippocampus has a definitive role in the short-time memory loss of Alzheimer’s disease, and is strongly affected by dementia and AD [48, 49, 4]. Therefore, our findings for hippocampus are in line with the literature data.

Apart from hippocampus, the temporal areas are present in numerous times in Table 1. In frontotemporal dementia, the shrinking of temporal areas are well-studied [50, 51]. In Alzheimer’s disease, the memory loss is also associated to temporal areas, and it is known that even in the early AD connection-loss happen in temporal lobes, especially in middle-temporal areas [52, 53].

It is interesting that in the first 45 vertices, given on Table 1, the Left Amygdala is present, while the right is not. This observation is in line with the literature data: in [54] it is shown that the neuronal loss is much higher in Left Amygdala than in right; in [55] volumetric studies have shown similar results for 38 subjects.

The correspondences with the earlier findings from the literature validate our methods and results.

### Vertices with strengthened connections in dementia

The data presented in Table 2 is more interesting in several aspects than that of Table 1. Table 2 shows the 45 vertices with the largest differences between healthy and demented weighted degrees, where the demented weighted degree is larger. That is, in these areas the connections became stronger in dementia than in the healthy graphs!

These results are not alone in the literature.

Subdivisions of the precuneus is present several times, both in the left and in the right hemispheres.

In work [56] was shown that the precuneus has increased functional connectivity in Alzheimer’s disease. Here, we have shown, that precuneus have increased structural connectivity in dementia, as well. [57] has shown larger activity of precuneus in early Alzheimer’s disease. Now we have shown higher structural connectivity as well.

Edges, connected to the insula are also become stronger in dementia by Table 2; similar findings for the increased gyrification of the insula was reported in [58]. In [59] similar findings were published. Now, we have shown a better structural connectivity of insula in dementia on a large sample.

## Supplementary Material

Supplement 1

## Figures and Tables

**Table 1:** Vertices with the largest weighted degree decrease ratio in dementia. Here the 45 vertices with the largest relative decrease are listed, the full table is given in the Appendix. In the D/H column the quotient of the averages of the weighted degrees are given, taken for the demented (D) and healthy (H) graphs.

Vertices with weakened connections in dementia													
Vertex		D/H ratio			Vertex		D/H ratio			Vertex		D/H ratio	
													
Left-Hippocampus_Fimbria			0.77		ctx-rh-fusiform_1			087		ctx-rh-inferiortemporal_5			0.90
Left-Hippocampus_HATA			0.78		ctx-lh-middletemporal_19			0.87		ctx-lh-temporalpole_2			0.90
Right-Hippocampus_Fimbria			0.81		Left-Central_Lateral.			0.88		ctx-rh-inferiortemporal_11			0.90
Right-Hippocampus_HATA			0.81		Left-Hippocampus_Paras.			0.88		ctx-rh-superiortemporal_1			0.90
Right-Hippocampus_Paras.			0.82		ctx-lh-middletemporal_9			0.88		ctx-lh-middletemporal_18			0.90
Left-Hippocampus			0.82		ctx-lh-entorhinal_1			0.89		ctx-rh-caudalmiddlefrontal_2			0.90
Right-Hippocampus			0.84		ctx-rh-middletemporal_19			0.89		ctx-lh-parahippocampal_5			0.90
Left-Hippocampus_Tail			0.85		ctx-lh-inferiortemporal_11			0.89		ctx-lh-middletemporal_1			0.90
Right-Hippocampus_Tail			0.85		Right-Ventral_Latero_Dorsal			0.89		ctx-lh-superiortemporal_1			0.91
ctx-lh-fusiform_1			0.86		Left-Ventral_Latero_Dorsal			0.89		ctx-lh-inferiortemporal_16			0.91
ctx-lh-parahippocampal_4			0.86		Left-Amygdala			0.89		ctx-rh-parahippocampal_5			0.91
ctx-rh-parahippocampal_4			0.87		ctx-lh-middletemporal_2			0.89		ctx-lh-inferiortemporal_5			0.91
ctx-lh-entorhinal_2			0.87		ctx-lh-inferiortemporal_1			0.90		ctx-rh-inferiortemporal_16			0.91
ctx-rh-entorhinal_2			0.87		ctx-rh-middletemporal_18			0.90		ctx-rh-middletemporal_9			0.91
ctx-rh-entorhinal_1			0.87		ctx-rh-middletemporal_5			0.90		ctx-rh-superiortemporal_10			0.91
													
Paras. = Parasubiculum													
Left-Central_Lateral.=		Left-Central_Lateral.-Lateral_Posterior-Medical_Pulvinar											

**Table 2:** Vertices with the largest relative weighted degree increase in dementia. Here the 45 vertices with the largest increase in the quotients are listed, the full table is given in the Appendix. In the D/H column the quotient of the averages of the weighted degrees are given, taken for the demented (D) and healthy (H) graphs.

Vertex		D/H ratio		Vertex		D/H ratio			Vertex		D/H ratio	
												
Brain_Stem-SCP		1.06		ctx-lh-precentral_4			1.07		ctx-lh-paracentral_8			1.08
ctx-rh-transversetemporal_3		1.06		ctx-lh-insula_13			1.07		ctx-lh-insula_1			1.08
ctx-rh-postcentral_29		1.06		ctx-rh-supramarginal_17			1.07		ctx-rh-superiortemporal_14			1.08
ctx-rh-pericalcarine_1		1.06		ctx-rh-postcentral_2			1.07		ctx-lh-cuneus_8			1.08
ctx-rh-precuneus_3		1.06		ctx-rh-paracentral_10			1.07		ctx-lh-precentral_16			1.08
ctx-lh-precentral_27		1.06		ctx-rh-paracentral_5			1.07		ctx-lh-precentral_22			1.08
ctx-lh-medialobitofrontal_11		1.06		ctx-rh-paracentral_8			1.07		ctx-lh-parstriangularis_8			1.08
ctx-rh-postcentral_19		1.06		ctx-rh-precuneus_1			1.07		ctx-lh-precentral_23			1.08
ctx-lh-pericalcarine_2		1.06		ctx-rh-postcentral_14			1.07		ctx-rh-insula_13			1.09
ctx-rh-postcentral_7		1.06		ctx-lh-precentral_33			1.07		ctx-lh-precentral_29			1.09
ctx-lh-postcentral_14		1.06		ctx-lh-pericalcarine_5			1.07		ctx-lh-paracentral_5			1.09
ctx-rh-precentral_33		1.06		ctx-lh-postcentral_19			1.07		ctx-lh-precuneus_1			1.10
ctx-lh-parsopercularis_2		1.06		ctx-lh-postcentral_2			1.07		ctx-lh-precuneus_3			1.10
ctx-lh-supramarginal_17		1.06		ctx-lh-pericalcarine_1			1.07		ctx-lh-precentral_24			1.10
ctx-lh-insula_3		1.06		ctx-lh-paracentral_7			1.07		Brain_Stem-Medulla			1.10

## Data Availability

The braingraphs are available under Section A at the site https://braingraph.org/cms/download-pit-group-connectomes/.
