# Supplementary material for: On the Increased and Decreased Connectivity of the Demented Human Brain
Source: ArXiv. 2026 Sep 9:arXiv:2607.05654v2. Originally published 2026 Jul 6. Preprint. [Version 2] (PMC13370593)
Supplement: Supplementary file 1 [file NIHPP2607.05654V2-supplement-1.pdf]

Appendix

Here we describe the node- and edge attributes in the GraphML files published in `https://braingraph.org/cms/download-pit-group-connectomes/`.

Node attributes

The node attributes in the GraphML files include the following values:

- dn_region can be cortical or subcortical
- dn_position_x, dn_position_y, dn_position_z the coordinates of a vertex. In a few cases, mostly in the substructures of the hippocampus, their values are missing if the substructure is not identified reliably. In that case, the NAN abbreviation (not a number) is given there.
- dn_name the corresponding anatomical name; it is either identical to or a refinement of the fsname attribute.
- dn_hemisphere left or right
- dn_fsname the corresponding anatomical area name of the node in FreeSurfer.
- dn_multiscaleID numerical node ID

  When, in lower resolutions, the sub-partitioning of small areas, mostly subcortical structures, cannot be distinguished from each other (e.g., the finer partitioning of the hippocampus), then the coordinate fields contain the word "nan".

Edge attributes

The edge attributes in the GraphML files include the following quantities:

- number_of_fibers, corresponding to an edge.
- fiber_length mean, median, and standard deviation (std) of the fiber lengths in mm, corresponding to an edge.
- fiber_density, normalized_fiber_density, fiber_proportion: Since fibers (or streamlines) can not always be tracked reliably in tractography algorithms, and since fibers may start or end erroneously in white matter during tractography (which is possible only in gray matter anatomically), some authors prefer to use fiber density quantities instead of or besides fiber numbers [73].
- The following quantities are related to the image reconstruction method SHORE: Simple harmonic oscillator based reconstruction and estimation [74, 75, 76]:
- shore_rtop_signal: RTOP: Return-to-Origin Probability [77].
- shore_msd: MSD: Mean Squared Displacement, with standard deviation (std), mean and median values; [78, 79]
- shore_gfa: GFA: derived Generalized Fractional Anisotropy (GFA) with standard deviation (std), mean and median values [80];

Demented-healthy change for all vertices

The following Table S1 contains the averaged fiber-number-weighted vertex degree for healthy and demented subjects. In the computation, we used graphs from 351 demented and 624 healthy subjects, where “demented” includes graphs with corresponding subjects who were not diagnosed as “demented” at the time of MRI recording but were later diagnosed in later sessions. The table contains 1044 rows, ordered by increasing D/H ratio. We considered 1058-vertex graphs; the missing 14 rows correspond to 14 vertices that were not connected to any edges, so we did not list those zero-degree vertices here. The p-values indicated here are not multiple-test corrected (for multiple-test correction, refer to the ”Statistical remarks ” subsection in the ”Methods” section).

Name	Demented	Healthy	D/H Ratio	p-value
Left-Hippocampus_Fimbria	102.1	132.5	0.77	2.64E-21
Left-Hippocampus_HATA	53.0	67.7	0.78	2.51E-15
Right-Hippocampus_Fimbria	94.0	116.3	0.81	1.91E-14
Right-Hippocampus_HATA	55.7	68.9	0.81	1.98E-12
Right-Hippocampus_Parasubiculum	80.2	98.0	0.82	1.72E-10
Left-Hippocampus	3177.6	3854.3	0.82	3.08E-31
Right-Hippocampus	3377.7	4011.8	0.84	8.35E-28
Left-Hippocampus_Tail	1027.1	1211.4	0.85	8.41E-27
Right-Hippocampus_Tail	1019.6	1194.7	0.85	2.89E-29
ctx-lh-fusiform_1	458.1	530.2	0.86	1.02E-10
ctx-lh-parahippocampal_4	532.9	616.4	0.86	1.20E-10
ctx-rh-parahippocampal_4	416.6	481.1	0.87	9.77E-12
ctx-lh-entorhinal_2	615.7	710.4	0.87	2.64E-10
ctx-rh-entorhinal_2	618.7	713.1	0.87	1.69E-12
ctx-rh-entorhinal_1	724.1	832.1	0.87	1.47E-13

ctx-rh-fusiform_1	558.2	641.2	0.87	3.66E-10
ctx-lh-middletemporal_19	597.1	684.1	0.87	1.25E-12
Left-Central_Lateral-Lateral_Posterior-Medial_Pulvinar	991.8	1131.2	0.88	8.33E-18
Left-Hippocampus_Parasubiculum	76.9	87.6	0.88	2.09E-05
ctx-lh-middletemporal_9	616.1	699.0	0.88	3.90E-13
ctx-lh-entorhinal_1	765.5	864.4	0.89	2.33E-10
ctx-rh-middletemporal_19	528.8	595.9	0.89	1.57E-10
ctx-lh-inferiortemporal_11	524.5	590.5	0.89	2.41E-10
Right-Ventral_Latero_Dorsal	1293.0	1452.5	0.89	3.64E-21
Left-Ventral_Latero_Dorsal	1384.3	1553.0	0.89	2.88E-20
Left-Amygdala	1482.9	1659.1	0.89	2.42E-21
ctx-lh-middletemporal_2	498.9	558.1	0.89	8.61E-09
ctx-lh-inferiortemporal_1	474.5	529.1	0.90	1.60E-08
ctx-rh-middletemporal_18	489.9	545.9	0.90	7.44E-07
ctx-rh-middletemporal_5	342.3	381.3	0.90	5.45E-07
ctx-rh-inferiortemporal_5	655.2	729.8	0.90	8.48E-09
ctx-lh-temporalpole_2	695.4	774.4	0.90	4.99E-13
ctx-rh-inferiortemporal_11	626.5	697.3	0.90	1.13E-07
ctx-rh-superiortemporal_1	420.2	467.6	0.90	5.86E-08
ctx-lh-middletemporal_18	515.5	571.3	0.90	1.18E-06
ctx-rh-caudalmiddlefrontal_2	577.2	639.6	0.90	5.91E-09
ctx-lh-parahippocampal_5	526.7	583.6	0.90	9.73E-13
ctx-lh-middletemporal_1	396.2	438.9	0.90	4.99E-07
ctx-lh-superiortemporal_1	454.5	501.4	0.91	1.40E-06
ctx-lh-inferiortemporal_16	564.5	622.4	0.91	1.08E-05
ctx-rh-parahippocampal_5	513.0	564.9	0.91	1.46E-10
ctx-lh-inferiortemporal_5	683.2	750.1	0.91	1.94E-07
ctx-rh-inferiortemporal_16	715.3	785.1	0.91	4.30E-06
ctx-rh-middletemporal_9	679.5	745.7	0.91	1.05E-09
ctx-rh-superiortemporal_10	319.3	350.1	0.91	5.65E-05
ctx-rh-middletemporal_2	496.8	544.3	0.91	1.56E-06
ctx-rh-superiorfrontal_29	461.6	505.1	0.91	2.23E-11
ctx-rh-temporalpole_2	690.5	754.9	0.91	7.82E-08
Right-Accumbens_area	1380.9	1508.9	0.92	4.03E-13
ctx-lh-superiorfrontal_29	452.8	494.7	0.92	7.78E-12
ctx-lh-middletemporal_7	361.6	394.4	0.92	7.17E-06
ctx-lh-inferiortemporal_9	373.1	406.7	0.92	3.88E-05
ctx-lh-fusiform_10	633.9	690.2	0.92	8.65E-06
ctx-rh-fusiform_3	512.3	556.3	0.92	3.50E-06
ctx-rh-superiorfrontal_7	621.2	674.1	0.92	8.31E-08
ctx-rh-inferiortemporal_8	389.5	422.0	0.92	2.97E-04
ctx-rh-caudalmiddlefrontal_9	925.7	1002.3	0.92	1.24E-07
ctx-lh-temporalpole_3	335.1	362.6	0.92	3.94E-05
ctx-rh-temporalpole_3	319.8	345.7	0.93	2.10E-04
ctx-rh-lateralorbitofrontal_4	556.1	600.2	0.93	6.57E-07
Right-Amygdala	1577.1	1701.8	0.93	5.60E-12
ctx-rh-inferiortemporal_12	510.3	550.6	0.93	2.19E-04
Right-Central_Lateral-Lateral_Posterior-Medial_Pulvinar	868.3	936.2	0.93	3.31E-07
ctx-lh-medialorbitofrontal_1	687.5	741.0	0.93	1.35E-06
Left-Accumbens_area	1358.1	1463.3	0.93	2.36E-10

ctx-lh-middletemporal_5	431.8	464.9	0.93	8.91E-04
ctx-lh-middletemporal_6	412.6	443.9	0.93	5.10E-05
ctx-rh-lateralorbitofrontal_7	581.0	624.3	0.93	3.07E-07
ctx-lh-superiorfrontal_9	553.1	594.3	0.93	4.14E-08
ctx-rh-fusiform_14	793.1	851.8	0.93	2.03E-06
ctx-rh-medialorbitofrontal_10	741.5	795.9	0.93	3.27E-04
ctx-rh-rostralmiddlefrontal_2	749.0	803.7	0.93	3.11E-05
Left-Ventral_Latero_Ventral	1855.8	1990.2	0.93	2.32E-09
ctx-rh-inferiortemporal_3	524.9	562.5	0.93	1.23E-03
ctx-rh-inferiortemporal_7	741.3	793.5	0.93	2.29E-04
ctx-lh-superiorfrontal_4	541.5	579.5	0.93	6.06E-06
ctx-lh-lateralorbitofrontal_7	591.7	633.1	0.93	3.16E-07
ctx-rh-lateralorbitofrontal_3	640.8	685.5	0.93	1.70E-05
ctx-rh-superiorfrontal_20	596.7	638.3	0.93	3.00E-07
ctx-rh-temporalpole_1	319.9	342.2	0.93	4.12E-03
ctx-lh-lateralorbitofrontal_15	374.6	400.7	0.94	6.05E-05
ctx-rh-caudalmiddlefrontal_4	542.1	579.7	0.94	1.70E-04
ctx-lh-inferiortemporal_14	685.8	733.3	0.94	7.90E-06
ctx-lh-lateralorbitofrontal_4	641.2	685.2	0.94	8.89E-07
Right-Ventral_Latero_Ventral	1992.8	2129.5	0.94	4.67E-11
ctx-rh-precentral_10	717.6	766.5	0.94	2.56E-06
ctx-lh-fusiform_3	527.7	563.6	0.94	3.03E-04
ctx-rh-bankssts_6	479.5	511.8	0.94	4.16E-05
ctx-rh-middletemporal_11	387.8	413.9	0.94	1.96E-04
ctx-rh-inferiortemporal_9	363.8	388.2	0.94	2.67E-03
Brain_Stem-Pons	2725.2	2906.9	0.94	6.60E-05
ctx-lh-inferiortemporal_2	756.4	806.8	0.94	7.98E-04
ctx-lh-precuneus_14	644.2	686.9	0.94	4.81E-06
Left-Pulvinar	1363.6	1453.8	0.94	9.74E-09
ctx-lh-inferiortemporal_4	607.3	646.8	0.94	1.45E-03
ctx-lh-superiorfrontal_10	486.4	517.9	0.94	4.09E-07
ctx-lh-parstriangularis_4	470.3	500.7	0.94	7.70E-05
ctx-rh-inferiortemporal_1	560.6	596.7	0.94	1.23E-03
ctx-rh-superiortemporal_8	468.9	499.1	0.94	9.29E-04
Right-Pulvinar	1377.2	1465.3	0.94	2.26E-08
ctx-rh-fusiform_11	323.0	343.7	0.94	5.35E-04
ctx-rh-fusiform_7	388.9	413.7	0.94	1.42E-05
ctx-rh-precentral_4	616.6	655.7	0.94	7.79E-04
ctx-lh-inferiortemporal_10	818.5	870.3	0.94	2.07E-03
ctx-rh-rostralmiddlefrontal_1	624.5	663.7	0.94	1.52E-04
ctx-rh-rostralmiddlefrontal_15	650.4	691.2	0.94	1.14E-03
ctx-lh-lateralorbitofrontal_8	347.9	369.5	0.94	3.60E-04
ctx-lh-middletemporal_11	352.2	374.0	0.94	3.02E-03
ctx-lh-supramarginal_18	580.1	615.6	0.94	1.69E-04
ctx-lh-caudalmiddlefrontal_2	710.4	753.8	0.94	7.06E-05
ctx-rh-fusiform_10	665.4	705.9	0.94	2.15E-03
ctx-rh-inferiortemporal_10	594.5	630.6	0.94	3.25E-03
ctx-lh-middletemporal_3	554.8	588.0	0.94	2.75E-04
ctx-lh-superiortemporal_20	702.8	744.6	0.94	5.52E-04
ctx-rh-fusiform_6	383.1	405.9	0.94	6.19E-04

ctx-lh-inferiortemporal_3	582.0	616.3	0.94	6.02E-03
ctx-rh-medialorbitofrontal_3	699.6	740.3	0.94	1.96E-03
ctx-rh-lateralorbitofrontal_14	708.4	749.6	0.95	2.50E-04
ctx-rh-fusiform_13	760.5	804.6	0.95	4.38E-05
ctx-lh-medialorbitofrontal_3	638.0	675.0	0.95	1.93E-03
ctx-lh-rostralmiddlefrontal_1	580.4	613.9	0.95	6.48E-04
ctx-lh-rostralmiddlefrontal_15	625.0	661.0	0.95	2.19E-04
ctx-rh-superiorfrontal_1	603.8	638.6	0.95	2.96E-04
ctx-rh-parstriangularis_4	624.9	660.6	0.95	3.04E-04
ctx-lh-lateralorbitofrontal_3	710.2	750.2	0.95	6.98E-05
ctx-rh-middletemporal_1	383.8	405.4	0.95	4.72E-03
ctx-rh-fusiform_4	593.1	626.4	0.95	1.29E-04
ctx-lh-lateralorbitofrontal_16	427.4	451.3	0.95	1.10E-03
ctx-rh-medialorbitofrontal_1	706.9	746.4	0.95	4.13E-04
ctx-lh-superiorfrontal_8	589.2	622.2	0.95	4.43E-07
ctx-lh-inferiortemporal_12	536.5	566.5	0.95	1.84E-03
Left-Hippocampus_Hippocampal_fissure	233.8	246.8	0.95	5.87E-02
ctx-lh-parsorbitalis_3	436.2	460.4	0.95	2.46E-04
ctx-rh-lateralorbitofrontal_13	509.0	537.1	0.95	1.05E-03
ctx-rh-bankssts_4	545.1	575.2	0.95	8.39E-05
ctx-rh-rostralmiddlefrontal_12	645.3	680.9	0.95	2.26E-04
ctx-rh-superiortemporal_20	518.5	547.0	0.95	5.11E-03
ctx-lh-medialorbitofrontal_9	638.1	673.1	0.95	9.94E-05
ctx-lh-middletemporal_17	448.7	473.3	0.95	3.47E-03
ctx-lh-medialorbitofrontal_5	427.1	450.2	0.95	3.31E-04
ctx-rh-superiortemporal_4	276.2	291.2	0.95	9.82E-03
ctx-rh-middletemporal_15	982.5	1035.4	0.95	3.65E-03
ctx-rh-parstriangularis_1	645.1	679.7	0.95	3.85E-04
ctx-lh-superiorfrontal_36	636.6	670.7	0.95	8.85E-07
ctx-rh-parsorbitalis_4	687.4	724.2	0.95	2.19E-04
ctx-rh-parsopercularis_7	566.7	597.0	0.95	3.16E-04
ctx-lh-caudalmiddlefrontal_9	1092.2	1150.5	0.95	3.56E-04
ctx-lh-inferiortemporal_8	370.0	389.7	0.95	2.47E-02
ctx-lh-lateraloccipital_7	649.3	683.8	0.95	2.21E-03
ctx-lh-bankssts_4	647.2	681.5	0.95	1.99E-04
ctx-rh-middletemporal_10	485.0	510.7	0.95	1.58E-03
ctx-lh-precuneus_11	616.8	649.4	0.95	5.35E-04
ctx-lh-superiorparietal_28	592.4	623.5	0.95	4.71E-03
ctx-rh-inferiortemporal_2	596.6	628.0	0.95	1.94E-02
ctx-lh-inferiorparietal_6	553.4	582.3	0.95	1.94E-04
ctx-rh-superiortemporal_7	522.6	549.7	0.95	2.50E-04
ctx-rh-middletemporal_7	393.4	413.8	0.95	2.86E-03
ctx-lh-superiorparietal_16	540.9	569.0	0.95	2.48E-03
ctx-lh-superiorfrontal_39	454.6	478.1	0.95	6.81E-05
ctx-rh-superiorfrontal_36	618.1	650.1	0.95	1.73E-05
ctx-lh-inferiorparietal_2	724.8	762.1	0.95	2.45E-04
ctx-rh-parsorbitalis_1	481.1	505.8	0.95	6.10E-04
ctx-rh-rostralmiddlefrontal_25	596.1	626.6	0.95	5.62E-03
ctx-lh-middletemporal_8	388.3	408.1	0.95	7.79E-03
ctx-rh-middletemporal_17	499.3	524.8	0.95	1.87E-03

ctx-rh-caudalanteriorcingulate_1	749.6	787.3	0.95	8.28E-05
ctx-lh-superiorfrontal_20	623.3	654.7	0.95	4.85E-06
ctx-rh-parsopercularis_6	605.5	635.9	0.95	1.69E-03
ctx-rh-lateralorbitofrontal_12	589.3	618.8	0.95	2.01E-03
ctx-lh-parahippocampal_1	538.6	565.5	0.95	4.85E-05
ctx-rh-superiorfrontal_32	607.1	637.5	0.95	1.07E-04
ctx-rh-middletemporal_16	762.4	800.3	0.95	3.29E-03
ctx-rh-parsorbitalis_3	632.9	664.2	0.95	2.09E-04
ctx-lh-caudalanteriorcingulate_1	521.1	546.7	0.95	5.72E-03
ctx-lh-middletemporal_13	435.6	457.0	0.95	9.77E-03
ctx-lh-inferiortemporal_7	736.2	772.3	0.95	5.85E-03
ctx-lh-superiortemporal_8	519.5	544.7	0.95	2.84E-03
ctx-lh-middletemporal_15	739.2	774.8	0.95	1.01E-02
ctx-rh-lateraloccipital_18	734.2	769.5	0.95	1.51E-03
ctx-rh-superiorfrontal_4	662.2	694.0	0.95	2.06E-03
ctx-rh-rostralmiddlefrontal_24	1088.4	1140.2	0.95	9.78E-04
ctx-rh-parsorbitalis_2	616.7	646.0	0.95	2.24E-03
ctx-rh-rostralmiddlefrontal_3	675.5	707.0	0.96	8.07E-03
ctx-rh-superiorfrontal_10	505.7	529.3	0.96	3.55E-04
ctx-lh-superiorparietal_21	348.8	365.1	0.96	1.22E-02
ctx-rh-insula_8	372.3	389.5	0.96	8.83E-03
ctx-rh-superiorparietal_28	605.6	633.7	0.96	1.13E-02
ctx-lh-superiorfrontal_32	620.0	648.7	0.96	5.79E-05
ctx-lh-rostralmiddlefrontal_2	800.0	836.5	0.96	3.99E-03
ctx-rh-parahippocampal_6	279.8	292.5	0.96	1.79E-02
ctx-lh-superiorfrontal_7	645.6	675.0	0.96	1.02E-03
ctx-lh-parsorbitalis_1	564.2	589.8	0.96	3.59E-04
ctx-lh-precentral_4	592.9	619.6	0.96	1.07E-02
ctx-lh-supramarginal_15	761.3	795.5	0.96	5.50E-03
ctx-lh-superiortemporal_10	376.5	393.3	0.96	2.67E-02
ctx-lh-inferiorparietal_4	705.4	736.7	0.96	4.18E-03
ctx-rh-superiorparietal_21	381.1	397.9	0.96	1.49E-02
ctx-rh-superiorfrontal_23	614.0	641.0	0.96	1.35E-03
ctx-lh-superiortemporal_18	655.0	683.6	0.96	2.94E-03
ctx-rh-inferiortemporal_14	647.1	675.4	0.96	1.83E-02
ctx-rh-parsopercularis_8	610.4	636.7	0.96	8.26E-03
ctx-rh-superiorfrontal_30	590.8	616.2	0.96	8.00E-03
ctx-lh-rostralmiddlefrontal_16	707.2	737.5	0.96	3.44E-03
Left-Ventral_Anterior	1398.2	1457.9	0.96	6.63E-04
ctx-rh-medialorbitofrontal_8	575.7	600.3	0.96	2.61E-03
ctx-lh-rostralmiddlefrontal_14	735.5	766.9	0.96	6.48E-03
ctx-rh-lateraloccipital_17	640.7	667.9	0.96	7.59E-03
ctx-rh-precentral_3	614.2	640.3	0.96	4.27E-03
ctx-rh-rostralmiddlefrontal_5	570.7	594.8	0.96	5.83E-03
ctx-rh-parahippocampal_1	481.7	502.0	0.96	2.05E-03
ctx-rh-inferiortemporal_15	852.6	888.5	0.96	1.38E-02
ctx-lh-precentral_2	599.2	624.3	0.96	4.23E-03
ctx-rh-inferiortemporal_4	522.3	544.3	0.96	4.46E-02
ctx-rh-medialorbitofrontal_9	562.7	586.3	0.96	7.57E-03
ctx-rh-rostralmiddlefrontal_17	596.7	621.7	0.96	1.43E-02

ctx-lh-rostralanteriorcingulate_4	885.0	921.6	0.96	8.38E-04
ctx-rh-superiortemporal_19	544.1	566.6	0.96	3.67E-03
ctx-rh-lateraloccipital_22	601.3	626.2	0.96	1.20E-02
ctx-rh-rostralmiddlefrontal_16	690.8	719.3	0.96	2.71E-02
ctx-rh-middletemporal_13	518.9	540.3	0.96	3.07E-02
ctx-rh-rostralmiddlefrontal_19	797.6	830.4	0.96	1.23E-02
Right-Ventral_Anterior	1354.9	1410.6	0.96	2.38E-06
ctx-rh-lateralorbitofrontal_10	463.1	482.1	0.96	5.48E-03
ctx-lh-rostralmiddlefrontal_25	617.4	642.6	0.96	1.13E-02
ctx-rh-medialorbitofrontal_4	406.6	423.1	0.96	1.14E-02
ctx-lh-superiorparietal_17	553.2	575.6	0.96	1.74E-02
ctx-lh-insula_2	420.6	437.6	0.96	9.61E-03
ctx-lh-inferiorparietal_5	466.6	485.4	0.96	1.37E-02
ctx-rh-superiorfrontal_8	595.9	619.7	0.96	1.05E-03
ctx-rh-lateraloccipital_6	641.8	667.3	0.96	1.23E-02
ctx-lh-lateralorbitofrontal_13	580.2	603.2	0.96	1.05E-02
ctx-lh-middletemporal_10	409.5	425.6	0.96	3.75E-02
ctx-lh-rostralmiddlefrontal_5	642.8	668.1	0.96	4.81E-03
ctx-rh-supramarginal_15	602.7	626.4	0.96	1.42E-02
ctx-rh-medialorbitofrontal_7	485.6	504.5	0.96	2.00E-02
ctx-lh-inferiorparietal_19	550.4	571.8	0.96	1.06E-02
ctx-rh-medialorbitofrontal_5	389.5	404.5	0.96	1.24E-02
ctx-rh-superiorfrontal_9	610.4	633.8	0.96	7.45E-03
ctx-lh-superiorparietal_2	423.5	439.6	0.96	2.01E-02
ctx-rh-caudalmiddlefrontal_7	636.5	660.6	0.96	2.14E-02
ctx-lh-rostralmiddlefrontal_27	500.4	519.3	0.96	1.14E-02
ctx-lh-rostralmiddlefrontal_19	730.1	757.5	0.96	1.84E-02
ctx-lh-inferiorparietal_7	480.1	498.1	0.96	8.68E-03
ctx-rh-superiorparietal_25	594.4	616.7	0.96	2.34E-02
ctx-rh-rostralanteriorcingulate_4	587.9	609.9	0.96	9.08E-03
ctx-lh-precentral_6	625.9	649.3	0.96	1.17E-02
ctx-rh-parstriangularis_2	722.1	748.9	0.96	1.21E-02
ctx-lh-superiorfrontal_15	556.5	577.2	0.96	2.86E-04
ctx-rh-parstriangularis_6	546.4	566.7	0.96	3.40E-02
ctx-rh-rostralmiddlefrontal_13	590.7	612.6	0.96	1.30E-02
ctx-rh-bankssts_5	732.7	759.8	0.96	5.64E-03
ctx-rh-bankssts_3	539.7	559.6	0.96	1.73E-02
ctx-rh-middletemporal_8	747.0	774.5	0.96	2.71E-02
ctx-lh-medialorbitofrontal_8	600.1	622.1	0.96	1.83E-02
ctx-lh-rostralmiddlefrontal_3	693.2	718.4	0.96	2.71E-02
ctx-lh-rostralmiddlefrontal_11	455.8	472.3	0.97	3.10E-02
ctx-lh-lateralorbitofrontal_14	835.0	865.2	0.97	1.04E-02
ctx-lh-frontalpole_2	120.9	125.2	0.97	1.68E-01
ctx-rh-parstriangularis_5	671.6	695.8	0.97	4.35E-03
ctx-lh-lateralorbitofrontal_2	694.2	719.0	0.97	1.58E-02
ctx-lh-precentral_10	860.7	891.3	0.97	8.63E-03
ctx-lh-parsopercularis_3	522.3	540.8	0.97	6.82E-03
ctx-rh-superiorfrontal_15	563.1	583.1	0.97	4.91E-03
ctx-rh-superiorfrontal_12	580.5	601.0	0.97	5.68E-03
ctx-lh-middletemporal_16	647.9	670.8	0.97	3.03E-02

ctx-rh-superiorfrontal_39	460.7	476.8	0.97	1.04E-02
ctx-lh-superiorparietal_25	641.1	663.5	0.97	2.78E-02
ctx-rh-precentral_18	795.4	823.1	0.97	3.76E-02
ctx-lh-insula_12	636.7	658.8	0.97	1.45E-02
ctx-rh-lateraloccipital_16	758.8	785.0	0.97	3.32E-02
ctx-rh-precentral_31	509.5	527.1	0.97	4.24E-02
ctx-lh-superiorfrontal_1	631.9	653.7	0.97	1.44E-02
ctx-lh-superiorfrontal_21	580.7	600.4	0.97	3.95E-02
ctx-rh-fusiform_5	425.7	440.1	0.97	5.68E-02
ctx-lh-precuneus_15	697.3	720.7	0.97	3.15E-02
ctx-rh-precuneus_13	659.3	681.5	0.97	1.18E-02
ctx-lh-superiorfrontal_37	578.1	597.4	0.97	1.60E-02
ctx-lh-caudalmiddlefrontal_4	622.2	643.0	0.97	3.67E-02
ctx-rh-parsopercularis_1	625.3	646.2	0.97	1.19E-02
ctx-lh-postcentral_24	199.2	205.8	0.97	3.17E-02
ctx-rh-precuneus_14	689.9	712.8	0.97	4.18E-02
ctx-rh-rostralanteriorcingulate_2	955.4	987.0	0.97	5.91E-03
ctx-rh-inferiorparietal_16	497.4	513.6	0.97	8.96E-02
ctx-rh-superiorfrontal_33	638.5	659.4	0.97	4.76E-02
ctx-rh-postcentral_25	484.7	500.5	0.97	1.49E-02
ctx-lh-inferiorparietal_17	563.2	581.5	0.97	4.53E-02
ctx-lh-precuneus_13	676.3	698.2	0.97	8.23E-03
ctx-lh-fusiform_4	601.3	620.7	0.97	1.63E-02
ctx-lh-parsopercularis_8	933.9	963.9	0.97	2.23E-02
ctx-rh-rostralmiddlefrontal_6	778.6	803.7	0.97	4.32E-02
ctx-lh-superiortemporal_19	480.5	495.9	0.97	5.12E-02
ctx-rh-precentral_6	594.1	613.1	0.97	3.47E-02
ctx-rh-caudalanteriorcingulate_2	547.6	565.2	0.97	1.17E-02
ctx-rh-fusiform_8	581.3	599.9	0.97	3.14E-02
ctx-lh-superiorfrontal_30	615.4	635.0	0.97	2.45E-02
ctx-lh-precuneus_18	642.7	663.1	0.97	1.87E-02
ctx-rh-lateraloccipital_11	795.4	820.7	0.97	2.61E-02
ctx-rh-inferiorparietal_15	619.0	638.5	0.97	7.44E-02
ctx-rh-rostralmiddlefrontal_8	445.5	459.6	0.97	5.35E-02
ctx-lh-rostralanteriorcingulate_2	1226.5	1264.9	0.97	3.89E-03
ctx-lh-rostralmiddlefrontal_9	969.9	1000.1	0.97	4.20E-02
ctx-lh-medialorbitofrontal_7	573.0	590.8	0.97	2.18E-02
ctx-rh-lateralorbitofrontal_1	600.5	619.2	0.97	4.43E-02
ctx-lh-superiorparietal_27	640.6	660.5	0.97	4.20E-02
ctx-lh-parahippocampal_6	275.8	284.3	0.97	1.52E-01
ctx-rh-cuneus_3	694.5	715.7	0.97	2.23E-02
ctx-rh-caudalanteriorcingulate_4	527.2	543.4	0.97	1.18E-02
ctx-lh-precentral_18	871.6	898.1	0.97	5.28E-02
ctx-rh-precentral_36	498.1	513.2	0.97	5.10E-02
ctx-rh-supramarginal_20	607.1	625.5	0.97	8.11E-02
ctx-lh-fusiform_13	623.8	642.6	0.97	4.38E-02
ctx-lh-parsopercularis_4	785.7	809.4	0.97	3.41E-02
ctx-lh-parsorbitalis_4	562.4	579.3	0.97	5.80E-02
ctx-lh-superiortemporal_15	638.5	657.4	0.97	1.67E-02
ctx-rh-superiortemporal_3	217.6	224.1	0.97	1.09E-01

ctx-lh-superiorparietal_18	704.4	725.2	0.97	7.24E-02
ctx-rh-precentral_2	613.9	631.9	0.97	5.67E-02
ctx-lh-superiorfrontal_40	562.2	578.5	0.97	4.73E-02
ctx-rh-inferiortemporal_13	1089.8	1121.5	0.97	5.07E-02
ctx-rh-cuneus_7	679.3	699.0	0.97	4.95E-02
ctx-rh-superiorfrontal_3	666.9	686.1	0.97	1.11E-01
ctx-lh-middletemporal_12	702.7	722.8	0.97	5.53E-02
ctx-lh-parsopercularis_6	585.3	602.0	0.97	6.87E-02
ctx-lh-parstriangularis_6	233.4	240.1	0.97	1.40E-01
ctx-rh-rostralmiddlefrontal_4	566.9	582.9	0.97	8.75E-02
ctx-rh-superiorparietal_27	629.7	647.4	0.97	7.65E-02
ctx-rh-superiorparietal_8	580.1	596.4	0.97	1.04E-01
ctx-rh-superiorfrontal_35	667.8	686.5	0.97	4.66E-02
ctx-lh-bankssts_6	511.2	525.5	0.97	7.32E-02
ctx-rh-inferiorparietal_5	495.3	509.1	0.97	8.69E-02
ctx-lh-superiorparietal_20	530.9	545.6	0.97	7.97E-02
ctx-rh-parsopercularis_3	448.4	460.8	0.97	5.37E-02
ctx-rh-rostralmiddlefrontal_11	569.4	585.1	0.97	7.76E-02
ctx-lh-rostralmiddlefrontal_13	469.4	482.3	0.97	7.03E-02
ctx-rh-parsopercularis_4	547.5	562.6	0.97	6.03E-02
ctx-lh-superiorfrontal_5	703.7	722.9	0.97	4.30E-02
ctx-rh-lateralorbitofrontal_17	468.2	481.0	0.97	5.65E-02
ctx-lh-superiortemporal_12	538.6	553.3	0.97	5.64E-02
ctx-lh-superiorfrontal_12	618.7	635.5	0.97	1.51E-02
ctx-lh-superiorparietal_9	896.6	920.6	0.97	6.58E-02
ctx-rh-lingual_8	672.5	690.5	0.97	8.67E-02
ctx-rh-supramarginal_9	720.8	740.1	0.97	1.31E-01
ctx-lh-precentral_3	603.8	619.9	0.97	4.63E-02
ctx-lh-lateraloccipital_17	654.0	671.2	0.97	9.34E-02
ctx-rh-superiorfrontal_21	540.2	554.3	0.97	1.15E-01
ctx-rh-posteriorcingulate_9	438.6	450.0	0.97	1.89E-01
ctx-rh-superiorfrontal_2	671.9	689.2	0.97	8.81E-02
ctx-lh-inferiortemporal_15	906.9	930.3	0.97	1.17E-01
ctx-rh-superiorparietal_24	594.0	609.3	0.97	2.27E-01
ctx-lh-rostralmiddlefrontal_17	597.0	612.3	0.98	9.99E-02
ctx-rh-cuneus_2	382.5	392.3	0.98	1.40E-01
ctx-rh-middletemporal_12	967.0	991.7	0.98	7.54E-02
ctx-rh-caudalanteriorcingulate_5	652.5	668.7	0.98	4.56E-02
ctx-rh-superiorfrontal_18	659.5	675.9	0.98	9.56E-02
ctx-rh-rostralmiddlefrontal_14	798.0	817.5	0.98	1.23E-01
ctx-lh-rostralmiddlefrontal_8	434.6	445.2	0.98	1.24E-01
ctx-lh-inferiorparietal_11	578.3	592.4	0.98	9.94E-02
ctx-rh-superiortemporal_12	612.5	627.3	0.98	4.26E-02
ctx-rh-superiorparietal_2	466.2	477.5	0.98	1.23E-01
ctx-lh-supramarginal_20	671.1	687.1	0.98	1.31E-01
ctx-lh-fusiform_8	521.5	533.9	0.98	9.65E-02
ctx-rh-superiorparietal_17	471.6	482.8	0.98	1.53E-01
ctx-lh-rostralmiddlefrontal_12	695.1	711.6	0.98	8.15E-02
ctx-rh-inferiorparietal_10	682.3	698.4	0.98	1.42E-01
ctx-lh-parstriangularis_5	771.2	789.4	0.98	5.83E-02

ctx-rh-superiortemporal_16	572.2	585.7	0.98	5.97E-02
ctx-lh-lateralorbitofrontal_12	650.2	665.4	0.98	9.61E-02
ctx-lh-fusiform_16	495.0	506.6	0.98	3.09E-01
ctx-lh-rostralmiddlefrontal_6	709.4	726.0	0.98	1.33E-01
ctx-lh-insula_14	438.6	448.9	0.98	9.07E-02
ctx-rh-rostralanteriorcingulate_3	631.0	645.7	0.98	7.01E-02
ctx-lh-superiorfrontal_38	552.5	565.3	0.98	5.17E-02
ctx-rh-medialorbitofrontal_11	623.5	637.9	0.98	1.27E-01
ctx-rh-supramarginal_14	628.2	642.7	0.98	1.17E-01
ctx-rh-inferiortemporal_6	792.7	810.9	0.98	2.33E-01
ctx-lh-superiortemporal_2	446.8	457.1	0.98	1.36E-01
ctx-lh-supramarginal_1	815.0	833.6	0.98	5.05E-02
ctx-rh-fusiform_16	368.8	377.2	0.98	3.33E-01
ctx-lh-inferiortemporal_13	994.3	1016.9	0.98	1.14E-01
ctx-rh-inferiorparietal_18	912.9	933.7	0.98	1.85E-01
ctx-lh-lateraloccipital_23	681.2	696.6	0.98	1.32E-01
ctx-rh-rostralmiddlefrontal_7	646.7	661.3	0.98	2.37E-01
ctx-rh-posteriorcingulate_5	553.3	565.8	0.98	2.71E-01
ctx-rh-middletemporal_3	512.0	523.5	0.98	1.73E-01
ctx-rh-superiorfrontal_38	562.6	575.2	0.98	1.21E-01
ctx-rh-lateralorbitofrontal_5	634.0	648.2	0.98	1.59E-01
Right-Anterior	1629.6	1666.0	0.98	5.79E-02
ctx-rh-insula_12	628.5	642.4	0.98	1.28E-01
ctx-lh-temporalpole_1	545.6	557.5	0.98	2.86E-01
Left-Anterior	1709.1	1746.3	0.98	7.97E-02
ctx-rh-rostralmiddlefrontal_9	775.2	792.0	0.98	1.79E-01
ctx-rh-lateraloccipital_2	717.1	732.6	0.98	9.36E-02
ctx-rh-superiorfrontal_34	585.4	598.0	0.98	1.60E-01
ctx-rh-parsopercularis_5	413.3	422.1	0.98	1.29E-01
ctx-rh-supramarginal_3	621.7	635.0	0.98	1.62E-01
ctx-lh-caudalanteriorcingulate_4	472.6	482.6	0.98	9.27E-02
ctx-rh-precentral_1	598.6	611.2	0.98	1.63E-01
ctx-rh-bankssts_2	656.9	670.6	0.98	1.83E-01
ctx-lh-inferiorparietal_21	594.7	607.2	0.98	2.26E-01
ctx-lh-superiorfrontal_23	721.8	736.7	0.98	1.09E-01
ctx-rh-superiorparietal_5	639.0	652.2	0.98	1.80E-01
ctx-rh-superiortemporal_18	611.0	623.4	0.98	1.80E-01
ctx-rh-inferiorparietal_17	683.8	697.7	0.98	2.14E-01
ctx-rh-precuneus_15	690.4	704.4	0.98	2.26E-01
ctx-rh-postcentral_3	538.9	549.8	0.98	1.39E-01
ctx-lh-supramarginal_4	503.1	513.2	0.98	2.08E-01
ctx-lh-superiorfrontal_34	642.9	655.8	0.98	1.62E-01
ctx-rh-fusiform_9	978.9	998.4	0.98	1.30E-01
ctx-lh-superiortemporal_7	491.0	500.8	0.98	1.88E-01
ctx-lh-parstriangularis_1	655.6	668.6	0.98	1.33E-01
ctx-rh-superiortemporal_9	538.5	549.1	0.98	1.96E-01
ctx-lh-caudalmiddlefrontal_7	728.1	742.5	0.98	1.88E-01
ctx-lh-lateralorbitofrontal_1	494.6	504.3	0.98	1.92E-01
ctx-rh-superiorparietal_16	542.5	553.0	0.98	2.68E-01
ctx-lh-lateraloccipital_19	679.1	692.3	0.98	1.94E-01

ctx-rh-superiorfrontal_14	595.4	606.9	0.98	2.23E-01
ctx-lh-superiortemporal_3	245.3	250.0	0.98	2.53E-01
ctx-rh-rostralmiddlefrontal_22	784.2	798.7	0.98	3.17E-01
ctx-lh-supramarginal_3	742.7	756.4	0.98	1.96E-01
ctx-rh-insula_2	502.4	511.6	0.98	1.78E-01
ctx-rh-rostralmiddlefrontal_10	839.0	854.4	0.98	2.49E-01
ctx-rh-superiortemporal_17	403.9	411.2	0.98	3.09E-01
ctx-rh-supramarginal_8	807.3	821.9	0.98	3.93E-01
ctx-lh-precuneus_7	783.7	797.8	0.98	2.09E-01
ctx-rh-postcentral_18	445.7	453.7	0.98	2.57E-01
ctx-lh-medialorbitofrontal_10	776.9	790.8	0.98	3.72E-01
ctx-rh-cuneus_4	724.4	737.4	0.98	2.60E-01
ctx-rh-rostralanteriorcingulate_1	620.5	631.5	0.98	2.03E-01
ctx-rh-parstriangularis_3	495.4	504.1	0.98	2.35E-01
ctx-lh-inferiorparietal_1	555.7	565.4	0.98	2.46E-01
ctx-lh-supramarginal_10	749.1	762.2	0.98	2.73E-01
ctx-lh-superiorparietal_13	976.2	993.3	0.98	2.47E-01
ctx-lh-inferiorparietal_3	551.4	561.0	0.98	2.67E-01
ctx-rh-lateraloccipital_19	701.7	713.8	0.98	2.44E-01
ctx-lh-superiortemporal_17	416.9	424.1	0.98	2.90E-01
ctx-lh-rostralmiddlefrontal_20	544.5	553.9	0.98	2.25E-01
ctx-lh-posteriorcingulate_4	587.0	596.9	0.98	1.39E-01
ctx-rh-superiorparietal_18	728.4	740.8	0.98	3.21E-01
ctx-rh-superiorfrontal_40	566.5	576.1	0.98	2.58E-01
ctx-lh-bankssts_1	613.6	623.9	0.98	2.63E-01
ctx-rh-lingual_16	665.1	676.2	0.98	2.63E-01
ctx-rh-precuneus_17	513.8	522.4	0.98	1.99E-01
ctx-lh-caudalanteriorcingulate_3	616.4	626.5	0.98	4.07E-01
ctx-rh-postcentral_6	635.0	645.4	0.98	1.59E-01
ctx-rh-superiorfrontal_5	728.0	739.9	0.98	2.63E-01
ctx-rh-fusiform_2	951.2	966.6	0.98	1.90E-01
ctx-lh-middletemporal_14	544.1	552.8	0.98	3.69E-01
ctx-rh-lateraloccipital_7	656.4	666.9	0.98	3.20E-01
ctx-lh-superiorparietal_5	580.0	589.2	0.98	2.78E-01
ctx-rh-inferiorparietal_21	703.3	714.4	0.98	4.24E-01
ctx-lh-inferiorparietal_24	684.9	695.7	0.98	2.75E-01
ctx-rh-precentral_21	285.0	289.5	0.98	4.74E-01
ctx-rh-parstriangularis_7	634.5	644.5	0.98	3.45E-01
ctx-rh-precentral_35	413.8	420.3	0.98	2.76E-01
ctx-rh-lateraloccipital_1	611.1	620.6	0.98	2.94E-01
ctx-rh-precentral_28	462.2	469.4	0.98	3.20E-01
ctx-lh-precuneus_5	443.6	450.5	0.98	2.65E-01
ctx-rh-precuneus_18	661.7	671.8	0.98	2.67E-01
ctx-lh-inferiorparietal_25	871.3	884.6	0.98	3.45E-01
ctx-lh-superiortemporal_23	478.7	486.0	0.98	2.86E-01
Left-VentralDC	2718.2	2759.5	0.99	7.67E-02
ctx-rh-lateralorbitofrontal_2	469.3	476.4	0.99	3.26E-01
Left-Medio_Dorsal	1071.7	1088.0	0.99	2.60E-01
ctx-rh-caudalanteriorcingulate_6	428.9	435.4	0.99	2.76E-01
ctx-lh-lingual_11	604.9	614.1	0.99	2.99E-01

ctx-rh-postcentral_20	495.7	503.1	0.99	2.16E-01
ctx-lh-fusiform_15	496.1	503.4	0.99	2.84E-01
ctx-lh-bankssts_2	669.2	679.1	0.99	2.97E-01
ctx-rh-precuneus_11	617.9	627.0	0.99	3.89E-01
ctx-rh-rostralmiddlefrontal_23	636.9	646.2	0.99	4.10E-01
ctx-rh-cuneus_6	754.4	765.4	0.99	2.95E-01
ctx-rh-rostralmiddlefrontal_27	531.1	538.9	0.99	3.63E-01
ctx-lh-postcentral_25	605.7	614.5	0.99	2.34E-01
ctx-rh-postcentral_15	389.2	394.8	0.99	4.02E-01
ctx-rh-lateraloccipital_4	663.8	673.3	0.99	3.29E-01
ctx-lh-superiorparietal_26	503.1	510.2	0.99	3.66E-01
ctx-rh-inferiorparietal_13	859.9	872.1	0.99	4.27E-01
ctx-rh-inferiorparietal_12	779.7	790.6	0.99	4.39E-01
ctx-lh-bankssts_3	652.7	661.8	0.99	3.81E-01
ctx-lh-medialorbitofrontal_4	467.6	474.1	0.99	3.01E-01
ctx-rh-lateraloccipital_3	640.1	649.0	0.99	4.34E-01
ctx-rh-insula_9	783.0	793.8	0.99	2.82E-01
ctx-lh-precentral_1	611.5	619.9	0.99	3.33E-01
ctx-lh-inferiorparietal_10	494.4	501.1	0.99	4.06E-01
ctx-rh-postcentral_23	381.6	386.8	0.99	4.50E-01
ctx-lh-inferiorparietal_16	425.0	430.8	0.99	4.29E-01
ctx-rh-bankssts_1	768.8	779.2	0.99	3.62E-01
ctx-rh-parahippocampal_2	383.7	388.9	0.99	3.98E-01
ctx-rh-supramarginal_4	423.8	429.4	0.99	4.23E-01
ctx-rh-cuneus_5	715.1	724.5	0.99	3.66E-01
ctx-lh-precentral_35	422.8	428.4	0.99	3.02E-01
ctx-lh-lateralorbitofrontal_5	719.4	728.8	0.99	3.84E-01
ctx-lh-lateraloccipital_2	845.4	856.4	0.99	2.89E-01
ctx-rh-inferiorparietal_23	748.7	758.4	0.99	4.14E-01
ctx-lh-superiorparietal_19	531.3	538.1	0.99	4.44E-01
ctx-lh-postcentral_3	536.0	542.8	0.99	3.12E-01
ctx-lh-parsopercularis_1	623.4	631.3	0.99	3.39E-01
ctx-rh-superiortemporal_2	500.5	506.8	0.99	3.57E-01
ctx-rh-isthmuscingulate_6	552.5	559.4	0.99	3.24E-01
ctx-lh-postcentral_12	528.1	534.7	0.99	4.16E-01
ctx-lh-insula_9	748.3	757.4	0.99	2.80E-01
ctx-rh-precuneus_7	730.4	739.3	0.99	3.87E-01
ctx-rh-inferiorparietal_4	797.6	807.3	0.99	4.03E-01
ctx-lh-superiorparietal_22	761.2	770.4	0.99	5.08E-01
ctx-rh-rostralmiddlefrontal_26	468.1	473.8	0.99	4.79E-01
ctx-rh-caudalmiddlefrontal_5	669.5	677.5	0.99	4.61E-01
ctx-rh-fusiform_15	487.1	493.0	0.99	4.25E-01
ctx-lh-supramarginal_8	835.2	845.1	0.99	5.11E-01
ctx-lh-lateraloccipital_18	674.3	682.2	0.99	4.37E-01
ctx-lh-transversetemporal_1	716.8	725.2	0.99	2.99E-01
ctx-rh-lingual_5	581.4	588.2	0.99	3.87E-01
ctx-lh-lateraloccipital_5	935.5	946.3	0.99	4.84E-01
ctx-rh-superiorfrontal_37	623.9	631.1	0.99	4.85E-01
ctx-lh-superiorfrontal_2	436.2	441.1	0.99	4.67E-01
Left-Pallidum	5378.1	5437.8	0.99	1.72E-01

ctx-rh-inferiorparietal_25	759.7	768.1	0.99	4.95E-01
ctx-rh-pericalcarine_8	646.2	653.3	0.99	5.22E-01
ctx-lh-superiorfrontal_33	726.2	734.2	0.99	4.17E-01
Brain_Stem-Midbrain	3689.9	3730.4	0.99	2.60E-01
ctx-lh-fusiform_12	689.7	697.2	0.99	4.72E-01
ctx-lh-parstriangularis_2	714.7	722.4	0.99	4.39E-01
ctx-lh-superiorparietal_8	566.6	572.6	0.99	5.05E-01
ctx-lh-superiorparietal_4	330.5	333.9	0.99	5.97E-01
ctx-lh-isthmuscingulate_6	606.3	612.5	0.99	3.61E-01
ctx-rh-caudalmiddlefrontal_3	704.9	712.1	0.99	5.39E-01
ctx-rh-precuneus_19	525.8	531.2	0.99	4.73E-01
ctx-rh-superiorparietal_26	728.5	735.9	0.99	4.88E-01
ctx-rh-superiorparietal_1	751.1	758.6	0.99	4.76E-01
ctx-rh-superiorfrontal_19	646.6	653.1	0.99	4.76E-01
ctx-rh-postcentral_12	472.9	477.5	0.99	5.63E-01
ctx-rh-lateraloccipital_20	561.5	567.0	0.99	5.11E-01
ctx-lh-rostralanteriorcingulate_1	796.2	804.0	0.99	4.12E-01
ctx-rh-supramarginal_18	564.1	569.6	0.99	5.41E-01
ctx-lh-postcentral_6	669.0	675.5	0.99	3.67E-01
ctx-lh-fusiform_9	1039.8	1049.8	0.99	4.65E-01
ctx-rh-middletemporal_6	438.5	442.7	0.99	5.75E-01
ctx-lh-precentral_5	643.0	649.1	0.99	3.88E-01
ctx-lh-rostralmiddlefrontal_24	1086.3	1096.7	0.99	4.69E-01
ctx-lh-lingual_13	649.2	655.4	0.99	5.15E-01
ctx-lh-parsopercularis_7	630.1	636.1	0.99	5.27E-01
ctx-rh-paracentral_12	450.8	455.0	0.99	5.45E-01
ctx-lh-lingual_15	504.4	509.0	0.99	5.51E-01
ctx-lh-bankssts_5	827.6	834.8	0.99	5.04E-01
ctx-lh-precentral_28	385.5	388.9	0.99	5.77E-01
ctx-lh-inferiorparietal_8	676.7	682.5	0.99	5.61E-01
ctx-rh-postcentral_28	499.5	503.8	0.99	5.42E-01
ctx-rh-insula_7	373.0	376.2	0.99	5.85E-01
ctx-lh-insula_8	380.4	383.6	0.99	5.97E-01
ctx-lh-rostralmiddlefrontal_4	746.7	753.0	0.99	5.33E-01
ctx-lh-rostralmiddlefrontal_21	951.7	959.7	0.99	5.44E-01
ctx-lh-postcentral_1	577.9	582.7	0.99	5.39E-01
ctx-rh-superiortemporal_22	398.2	401.5	0.99	5.92E-01
ctx-lh-postcentral_17	537.5	541.9	0.99	5.85E-01
ctx-rh-superiorparietal_15	659.9	665.2	0.99	5.91E-01
ctx-rh-superiorparietal_6	528.0	532.0	0.99	5.92E-01
ctx-lh-rostralmiddlefrontal_22	729.8	735.2	0.99	6.38E-01
ctx-lh-inferiorparietal_14	413.6	416.6	0.99	7.31E-01
ctx-lh-medialorbitofrontal_6	518.1	522.0	0.99	6.42E-01
ctx-rh-isthmuscingulate_2	511.2	515.0	0.99	6.06E-01
ctx-lh-cuneus_6	762.8	768.4	0.99	6.04E-01
ctx-rh-caudalmiddlefrontal_1	918.9	925.6	0.99	6.07E-01
ctx-lh-supramarginal_9	959.9	966.9	0.99	5.95E-01
ctx-rh-posteriorcingulate_8	558.3	562.3	0.99	5.92E-01
ctx-lh-postcentral_23	447.4	450.5	0.99	6.89E-01
ctx-rh-superiorfrontal_6	613.8	618.1	0.99	6.80E-01

ctx-rh-superiortemporal_15	665.6	670.3	0.99	5.55E-01
ctx-rh-middletemporal_14	509.5	513.0	0.99	6.67E-01
ctx-rh-pericalcarine_6	581.7	585.8	0.99	6.32E-01
ctx-rh-lateraloccipital_12	718.3	723.1	0.99	6.48E-01
ctx-rh-lateraloccipital_15	738.0	742.9	0.99	6.67E-01
ctx-lh-superiortemporal_13	480.3	483.5	0.99	5.94E-01
ctx-lh-precuneus_21	577.8	581.7	0.99	6.02E-01
ctx-rh-pericalcarine_3	629.5	633.6	0.99	6.44E-01
ctx-lh-cuneus_7	624.2	628.2	0.99	6.52E-01
ctx-lh-inferiorparietal_22	402.7	405.3	0.99	7.08E-01
ctx-lh-lateralorbitofrontal_10	522.2	525.4	0.99	6.48E-01
ctx-lh-parsopercularis_5	619.3	623.2	0.99	5.97E-01
ctx-rh-lingual_14	572.7	576.3	0.99	7.09E-01
ctx-rh-fusiform_12	695.9	700.1	0.99	6.65E-01
ctx-rh-supramarginal_16	704.2	708.3	0.99	7.09E-01
ctx-lh-caudalanteriorcingulate_6	311.5	313.3	0.99	6.96E-01
ctx-lh-isthmuscingulate_2	533.8	536.8	0.99	6.75E-01
ctx-rh-precentral_14	558.8	561.9	0.99	7.22E-01
ctx-rh-lateraloccipital_9	654.5	658.2	0.99	7.34E-01
ctx-rh-precuneus_8	747.0	751.2	0.99	7.19E-01
ctx-lh-superiorfrontal_35	1168.6	1175.2	0.99	6.20E-01
ctx-rh-cuneus_1	539.3	542.3	0.99	6.86E-01
ctx-lh-lingual_8	644.1	647.7	0.99	7.27E-01
ctx-rh-lateraloccipital_8	945.1	950.3	0.99	6.83E-01
ctx-rh-lingual_11	560.1	563.2	0.99	7.37E-01
ctx-rh-inferiorparietal_11	707.5	711.3	0.99	7.41E-01
ctx-rh-postcentral_10	375.8	377.8	0.99	7.87E-01
ctx-lh-supramarginal_19	399.4	401.5	0.99	7.68E-01
ctx-lh-postcentral_18	493.8	496.3	0.99	7.13E-01
ctx-lh-lateraloccipital_14	536.7	539.4	0.99	7.54E-01
ctx-rh-lingual_13	590.7	593.6	1.00	7.33E-01
ctx-lh-caudalmiddlefrontal_1	1118.6	1124.0	1.00	6.90E-01
ctx-lh-postcentral_4	397.0	398.8	1.00	7.20E-01
ctx-lh-fusiform_14	868.3	872.2	1.00	7.39E-01
ctx-rh-rostralmiddlefrontal_18	720.4	723.6	1.00	8.08E-01
ctx-lh-rostralmiddlefrontal_10	960.7	964.8	1.00	7.69E-01
ctx-lh-isthmuscingulate_3	606.1	608.7	1.00	7.70E-01
ctx-lh-lateraloccipital_12	620.4	622.9	1.00	7.91E-01
ctx-rh-posteriorcingulate_2	524.8	526.6	1.00	7.97E-01
ctx-lh-superiortemporal_22	405.7	407.0	1.00	8.45E-01
ctx-rh-superiorfrontal_25	504.5	506.1	1.00	8.44E-01
ctx-lh-parsorbitalis_2	482.6	484.1	1.00	8.43E-01
ctx-rh-inferiorparietal_20	697.5	699.7	1.00	8.50E-01
ctx-lh-superiortemporal_4	350.0	351.0	1.00	8.61E-01
ctx-rh-parahippocampal_3	495.1	496.5	1.00	8.28E-01
Right-Hippocampus_Hippocampal_fissure	271.0	271.7	1.00	8.99E-01
ctx-rh-insula_4	378.6	379.5	1.00	8.61E-01
ctx-lh-postcentral_20	502.1	503.3	1.00	8.31E-01
ctx-rh-frontalpole_1	519.4	520.6	1.00	8.86E-01
ctx-lh-precuneus_17	387.5	388.4	1.00	8.62E-01

ctx-lh-paracentral_1	527.9	529.1	1.00	8.51E-01
ctx-rh-lateralorbitofrontal_16	450.5	451.6	1.00	8.80E-01
ctx-lh-postcentral_26	626.2	627.6	1.00	8.55E-01
ctx-lh-lateraloccipital_9	584.7	585.9	1.00	9.05E-01
ctx-lh-lateraloccipital_21	687.3	688.7	1.00	8.95E-01
ctx-lh-superiorparietal_6	346.3	346.9	1.00	9.04E-01
ctx-rh-posteriorcingulate_3	492.8	493.7	1.00	9.21E-01
ctx-lh-precentral_8	525.8	526.7	1.00	9.09E-01
ctx-lh-lateraloccipital_20	538.4	539.2	1.00	9.15E-01
ctx-rh-superiorfrontal_16	488.4	489.0	1.00	9.38E-01
ctx-lh-fusiform_7	411.1	411.7	1.00	9.27E-01
ctx-lh-superiorfrontal_14	567.0	567.6	1.00	9.41E-01
ctx-lh-superiortemporal_16	222.6	222.8	1.00	9.55E-01
ctx-rh-paracentral_11	575.6	576.2	1.00	9.46E-01
ctx-rh-lateraloccipital_23	772.2	772.9	1.00	9.49E-01
ctx-rh-precuneus_22	432.2	432.6	1.00	9.48E-01
ctx-rh-inferiorparietal_14	639.5	640.1	1.00	9.59E-01
ctx-rh-inferiorparietal_19	841.7	842.3	1.00	9.63E-01
ctx-rh-superiorfrontal_26	696.7	697.1	1.00	9.68E-01
ctx-rh-caudalmiddlefrontal_6	894.4	894.9	1.00	9.70E-01
ctx-lh-postcentral_22	513.0	513.2	1.00	9.76E-01
ctx-lh-supramarginal_14	658.2	658.4	1.00	9.82E-01
ctx-rh-superiorparietal_19	606.3	606.5	1.00	9.83E-01
ctx-lh-supramarginal_13	873.5	873.5	1.00	9.97E-01
ctx-rh-superiorparietal_9	824.9	824.9	1.00	9.98E-01
ctx-rh-precuneus_4	529.6	529.6	1.00	9.99E-01
ctx-lh-superiorfrontal_19	814.3	814.2	1.00	9.89E-01
ctx-rh-lateralorbitofrontal_6	711.6	711.5	1.00	9.88E-01
ctx-lh-lingual_5	860.1	859.9	1.00	9.80E-01
ctx-lh-parahippocampal_3	293.2	293.1	1.00	9.75E-01
ctx-rh-lingual_1	727.3	727.0	1.00	9.80E-01
ctx-lh-superiorparietal_7	673.2	672.9	1.00	9.75E-01
ctx-lh-posteriorcingulate_9	456.0	455.8	1.00	9.74E-01
ctx-lh-precuneus_8	784.5	784.0	1.00	9.61E-01
ctx-rh-precentral_34	500.2	499.8	1.00	9.65E-01
ctx-rh-lateraloccipital_5	769.9	769.2	1.00	9.62E-01
ctx-lh-lateraloccipital_6	604.4	603.9	1.00	9.54E-01
ctx-lh-fusiform_17	434.2	433.9	1.00	9.49E-01
ctx-rh-superiorparietal_23	538.1	537.7	1.00	9.60E-01
ctx-rh-precuneus_21	574.0	573.5	1.00	9.56E-01
ctx-rh-precentral_9	576.6	576.1	1.00	9.59E-01
ctx-rh-insula_14	423.8	423.4	1.00	9.48E-01
Right-Pallidum	5073.8	5068.3	1.00	8.97E-01
ctx-lh-superiorfrontal_6	668.6	667.8	1.00	9.36E-01
ctx-rh-precentral_13	634.4	633.6	1.00	9.48E-01
ctx-rh-posteriorcingulate_7	666.5	665.6	1.00	9.26E-01
ctx-rh-superiorparietal_7	574.6	573.9	1.00	9.37E-01
ctx-rh-supramarginal_13	710.6	709.6	1.00	9.44E-01
ctx-lh-precentral_25	857.9	856.7	1.00	9.12E-01
ctx-lh-supramarginal_2	237.0	236.6	1.00	9.38E-01

ctx-rh-lateralorbitofrontal_8	358.3	357.8	1.00	9.30E-01
ctx-lh-lingual_16	648.5	647.5	1.00	9.17E-01
ctx-lh-lateraloccipital_13	768.9	767.6	1.00	8.99E-01
ctx-lh-posteriorcingulate_2	480.7	479.8	1.00	8.88E-01
ctx-lh-parstriangularis_3	455.3	454.5	1.00	9.03E-01
ctx-rh-precentral_5	536.3	535.2	1.00	8.65E-01
ctx-rh-lingual_9	769.2	767.5	1.00	8.79E-01
ctx-lh-lateralorbitofrontal_17	546.1	544.9	1.00	8.71E-01
ctx-rh-caudalanteriorcingulate_3	716.9	715.3	1.00	8.81E-01
ctx-rh-supramarginal_10	643.2	641.7	1.00	8.90E-01
ctx-lh-inferiorparietal_9	593.2	591.8	1.00	8.76E-01
ctx-rh-superiorparietal_10	745.6	743.8	1.00	8.90E-01
ctx-rh-postcentral_26	544.9	543.4	1.00	8.55E-01
ctx-rh-superiorfrontal_22	754.9	752.9	1.00	8.62E-01
ctx-lh-rostralmiddlefrontal_23	569.2	567.7	1.00	8.56E-01
ctx-rh-superiorparietal_3	566.6	565.0	1.00	8.67E-01
ctx-rh-medialorbitofrontal_6	618.2	616.4	1.00	8.38E-01
ctx-lh-parahippocampal_2	288.9	288.1	1.00	8.64E-01
ctx-rh-rostralmiddlefrontal_21	916.7	914.0	1.00	8.64E-01
ctx-lh-precentral_21	352.4	351.3	1.00	8.61E-01
ctx-rh-superiorparietal_29	497.1	495.6	1.00	8.40E-01
ctx-lh-precentral_14	602.8	600.9	1.00	8.29E-01
ctx-rh-lateraloccipital_13	825.4	822.8	1.00	8.23E-01
ctx-lh-inferiorparietal_26	433.2	431.7	1.00	8.29E-01
ctx-rh-rostralmiddlefrontal_20	663.3	660.9	1.00	8.45E-01
ctx-rh-paracentral_1	433.0	431.5	1.00	8.23E-01
ctx-rh-inferiorparietal_26	463.8	462.1	1.00	8.47E-01
ctx-rh-lateraloccipital_14	525.3	523.4	1.00	8.38E-01
ctx-rh-inferiorparietal_8	684.4	681.9	1.00	8.23E-01
ctx-rh-superiortemporal_23	452.7	451.1	1.00	8.03E-01
ctx-lh-precuneus_19	493.3	491.3	1.00	7.57E-01
ctx-rh-posteriorcingulate_1	605.7	603.2	1.00	8.30E-01
ctx-rh-superiorparietal_4	383.0	381.4	1.00	8.13E-01
ctx-lh-precuneus_22	469.8	467.8	1.00	7.29E-01
ctx-rh-lateralorbitofrontal_11	475.8	473.7	1.00	7.68E-01
ctx-lh-inferiorparietal_12	753.2	749.8	1.00	7.76E-01
ctx-rh-lingual_15	612.5	609.7	1.00	7.96E-01
ctx-lh-postcentral_9	628.3	625.4	1.00	7.51E-01
ctx-lh-inferiortemporal_6	818.7	814.6	1.00	7.68E-01
Right-Putamen	9624.0	9575.3	1.01	5.04E-01
ctx-lh-superiorparietal_15	625.5	622.3	1.01	7.25E-01
ctx-rh-postcentral_11	468.2	465.8	1.01	7.04E-01
ctx-lh-posteriorcingulate_7	669.2	665.7	1.01	6.85E-01
ctx-rh-fusiform_17	549.7	546.7	1.01	7.27E-01
ctx-rh-postcentral_17	456.5	453.9	1.01	7.30E-01
ctx-lh-lateraloccipital_1	693.8	689.7	1.01	6.81E-01
ctx-rh-lingual_17	331.7	329.8	1.01	7.25E-01
ctx-lh-superiortemporal_25	586.1	582.6	1.01	6.84E-01
ctx-rh-superiorfrontal_11	706.4	701.9	1.01	6.96E-01
ctx-lh-superiorfrontal_18	665.9	661.6	1.01	6.36E-01

ctx-rh-superiortemporal_13	521.5	518.1	1.01	5.93E-01
ctx-rh-inferiorparietal_22	791.2	785.8	1.01	6.50E-01
ctx-lh-lingual_7	504.7	501.3	1.01	6.83E-01
ctx-lh-superiorfrontal_3	643.7	639.2	1.01	7.06E-01
ctx-lh-superiortemporal_24	442.9	439.9	1.01	6.77E-01
Left-Putamen	9510.7	9443.0	1.01	2.92E-01
ctx-lh-postcentral_16	430.2	427.2	1.01	5.68E-01
ctx-lh-isthmuscingulate_1	695.8	690.8	1.01	5.44E-01
ctx-lh-cuneus_2	404.4	401.5	1.01	6.49E-01
ctx-rh-supramarginal_7	715.2	709.9	1.01	5.41E-01
ctx-lh-lateraloccipital_11	776.4	770.7	1.01	5.58E-01
ctx-lh-rostralmiddlefrontal_7	732.3	726.8	1.01	6.53E-01
ctx-rh-precuneus_5	518.7	514.8	1.01	6.34E-01
ctx-lh-fusiform_6	384.3	381.3	1.01	6.91E-01
ctx-lh-rostralanteriorcingulate_3	769.0	763.1	1.01	5.54E-01
ctx-lh-inferiorparietal_23	343.4	340.7	1.01	6.63E-01
ctx-lh-pericalcarine_4	853.7	847.0	1.01	6.12E-01
ctx-rh-isthmuscingulate_3	597.5	592.7	1.01	6.13E-01
ctx-lh-postcentral_28	543.5	539.2	1.01	5.65E-01
ctx-lh-supramarginal_16	572.4	567.9	1.01	6.37E-01
ctx-lh-middletemporal_4	593.3	588.6	1.01	6.31E-01
ctx-rh-lingual_10	781.3	774.9	1.01	5.60E-01
ctx-rh-supramarginal_1	638.9	633.5	1.01	5.04E-01
ctx-lh-precentral_36	454.1	450.3	1.01	6.06E-01
ctx-lh-postcentral_10	398.4	394.9	1.01	6.54E-01
ctx-rh-middletemporal_4	515.5	511.0	1.01	5.72E-01
ctx-lh-precuneus_9	505.1	500.6	1.01	5.04E-01
ctx-rh-posteriorcingulate_6	654.2	648.3	1.01	5.61E-01
Right-Caudate	7015.9	6952.0	1.01	3.87E-01
ctx-lh-lateraloccipital_4	674.0	667.4	1.01	4.93E-01
ctx-lh-superiorfrontal_24	462.8	458.3	1.01	5.50E-01
ctx-rh-precuneus_6	506.0	501.0	1.01	5.09E-01
ctx-rh-posteriorcingulate_4	498.3	493.4	1.01	4.66E-01
ctx-rh-postcentral_24	499.6	494.6	1.01	4.86E-01
ctx-rh-superiorfrontal_27	540.5	535.2	1.01	5.61E-01
ctx-lh-precentral_34	548.4	543.0	1.01	5.44E-01
ctx-rh-precentral_8	512.4	507.2	1.01	4.95E-01
ctx-rh-superiorparietal_14	525.1	519.8	1.01	6.02E-01
ctx-lh-insula_5	519.6	514.3	1.01	4.84E-01
ctx-lh-superiortemporal_9	865.8	856.9	1.01	5.26E-01
ctx-lh-inferiorparietal_13	555.0	549.3	1.01	5.70E-01
ctx-rh-caudalmiddlefrontal_11	563.5	557.7	1.01	5.32E-01
ctx-rh-inferiorparietal_2	789.1	780.9	1.01	4.81E-01
ctx-lh-postcentral_15	529.1	523.6	1.01	5.04E-01
ctx-rh-isthmuscingulate_4	651.6	644.8	1.01	4.07E-01
ctx-rh-lingual_7	471.6	466.6	1.01	5.92E-01
ctx-rh-transversetemporal_1	561.6	555.7	1.01	3.82E-01
ctx-rh-superiortemporal_6	499.5	494.1	1.01	4.72E-01
ctx-rh-precuneus_23	546.9	540.9	1.01	4.36E-01
ctx-lh-lingual_3	697.7	690.0	1.01	4.45E-01

ctx-rh-inferiorparietal_1	578.6	572.1	1.01	5.15E-01
ctx-lh-cuneus_4	609.2	602.3	1.01	4.72E-01
ctx-rh-lingual_6	902.8	892.7	1.01	5.99E-01
ctx-rh-postcentral_4	406.6	401.9	1.01	3.95E-01
ctx-rh-isthmuscingulate_1	695.0	687.0	1.01	4.06E-01
ctx-rh-superiortemporal_25	414.7	409.9	1.01	4.19E-01
ctx-rh-isthmuscingulate_5	588.9	582.0	1.01	3.43E-01
ctx-lh-superiortemporal_21	469.9	464.3	1.01	4.32E-01
ctx-lh-fusiform_2	883.9	873.2	1.01	3.34E-01
ctx-lh-supramarginal_7	726.6	717.6	1.01	2.90E-01
ctx-rh-lingual_3	669.3	660.9	1.01	3.95E-01
ctx-rh-lateralorbitofrontal_15	390.1	385.2	1.01	4.50E-01
ctx-lh-pericalcarine_8	528.7	521.9	1.01	4.73E-01
ctx-lh-superiorfrontal_11	535.1	528.3	1.01	4.04E-01
ctx-rh-insula_5	545.3	538.3	1.01	3.84E-01
ctx-rh-lateraloccipital_21	739.5	730.0	1.01	4.06E-01
ctx-lh-precuneus_12	419.9	414.4	1.01	3.76E-01
ctx-rh-lateralorbitofrontal_9	470.1	463.9	1.01	3.75E-01
ctx-rh-superiorparietal_22	697.2	687.9	1.01	4.83E-01
ctx-lh-superiorfrontal_13	693.9	684.6	1.01	4.05E-01
ctx-rh-precentral_19	528.9	521.8	1.01	3.76E-01
ctx-lh-lingual_4	918.5	905.8	1.01	2.28E-01
ctx-rh-supramarginal_2	449.4	443.2	1.01	3.49E-01
ctx-lh-superiorfrontal_26	839.1	827.5	1.01	3.45E-01
ctx-lh-inferiorparietal_18	795.7	784.7	1.01	3.38E-01
ctx-lh-lingual_17	374.9	369.7	1.01	3.57E-01
ctx-rh-postcentral_16	416.5	410.7	1.01	3.24E-01
ctx-lh-lingual_10	609.2	600.6	1.01	2.76E-01
ctx-lh-superiorfrontal_22	684.5	674.8	1.01	3.35E-01
ctx-lh-insula_4	399.2	393.5	1.01	2.75E-01
ctx-lh-superiorparietal_1	1003.3	988.6	1.01	2.56E-01
ctx-lh-posteriorcingulate_5	602.8	593.9	1.01	3.96E-01
ctx-rh-caudalmiddlefrontal_8	572.6	564.1	1.01	3.92E-01
ctx-rh-superiortemporal_5	359.7	354.3	1.02	2.85E-01
ctx-lh-precentral_20	708.3	697.8	1.02	3.03E-01
ctx-lh-parstriangularis_7	483.6	476.2	1.02	3.40E-01
ctx-rh-superiorfrontal_28	724.3	713.2	1.02	3.93E-01
ctx-lh-lingual_14	528.4	520.2	1.02	3.46E-01
ctx-lh-superiortemporal_5	370.1	364.4	1.02	2.60E-01
ctx-rh-inferiorparietal_6	663.2	653.0	1.02	3.51E-01
ctx-lh-inferiorparietal_15	628.3	618.6	1.02	3.37E-01
ctx-rh-lateraloccipital_10	549.4	540.8	1.02	2.65E-01
ctx-lh-superiorfrontal_25	491.4	483.7	1.02	3.22E-01
ctx-rh-insula_10	598.0	588.6	1.02	2.67E-01
ctx-rh-precuneus_10	676.4	665.7	1.02	3.33E-01
ctx-lh-medialorbitofrontal_2	265.1	260.9	1.02	4.21E-01
ctx-rh-precuneus_12	535.3	526.6	1.02	2.72E-01
ctx-rh-pericalcarine_7	898.8	884.2	1.02	2.17E-01
ctx-rh-postcentral_8	548.1	539.1	1.02	3.02E-01
ctx-lh-caudalanteriorcingulate_2	511.7	503.2	1.02	2.12E-01

Right-VentralDC	2897.1	2848.7	1.02	6.07E-02
ctx-lh-fusiform_5	427.5	420.3	1.02	3.67E-01
ctx-lh-lateralorbitofrontal_11	371.3	365.1	1.02	2.93E-01
ctx-rh-superiorfrontal_17	805.5	791.9	1.02	2.28E-01
ctx-rh-superiorfrontal_31	686.6	675.0	1.02	2.85E-01
ctx-lh-superiorfrontal_28	661.3	650.0	1.02	2.52E-01
ctx-rh-inferiorparietal_9	622.5	611.8	1.02	3.34E-01
ctx-lh-lateraloccipital_8	674.0	662.4	1.02	2.57E-01
ctx-lh-superiorparietal_23	455.5	447.4	1.02	3.16E-01
ctx-lh-lingual_12	680.7	668.6	1.02	1.50E-01
ctx-lh-lateraloccipital_22	773.4	759.7	1.02	2.26E-01
ctx-rh-pericalcarine_4	939.6	922.9	1.02	2.47E-01
ctx-rh-inferiorparietal_7	534.0	524.5	1.02	2.43E-01
ctx-lh-superiorparietal_24	718.1	704.8	1.02	3.75E-01
ctx-rh-postcentral_1	544.6	534.5	1.02	2.18E-01
ctx-rh-insula_3	466.0	457.4	1.02	2.76E-01
ctx-lh-superiorfrontal_16	545.8	535.6	1.02	2.44E-01
ctx-rh-supramarginal_5	634.4	622.5	1.02	1.50E-01
ctx-lh-postcentral_11	589.7	578.7	1.02	1.50E-01
ctx-lh-supramarginal_12	611.8	600.2	1.02	1.83E-01
ctx-lh-rostralmiddlefrontal_18	717.7	704.1	1.02	2.51E-01
ctx-lh-superiorfrontal_27	594.8	583.5	1.02	1.48E-01
ctx-rh-inferiorparietal_3	680.6	667.7	1.02	2.36E-01
ctx-lh-caudalmiddlefrontal_6	885.1	868.3	1.02	1.62E-01
ctx-rh-superiortemporal_24	490.1	480.7	1.02	1.90E-01
ctx-rh-superiorparietal_13	798.6	783.3	1.02	3.01E-01
ctx-rh-precuneus_9	569.4	558.3	1.02	1.56E-01
ctx-rh-superiortemporal_21	668.7	655.5	1.02	1.44E-01
ctx-rh-precentral_25	832.7	815.9	1.02	2.09E-01
ctx-lh-frontalpole_1	534.9	524.1	1.02	2.01E-01
ctx-lh-pericalcarine_3	589.1	577.2	1.02	1.65E-01
ctx-lh-posteriorcingulate_1	654.9	641.4	1.02	2.89E-01
ctx-rh-lingual_4	1025.5	1004.1	1.02	8.14E-02
ctx-lh-inferiorparietal_20	499.4	488.8	1.02	2.63E-01
ctx-lh-lateraloccipital_16	713.1	698.0	1.02	1.59E-01
ctx-lh-precuneus_4	489.4	478.8	1.02	3.11E-01
ctx-lh-precentral_30	513.2	502.0	1.02	1.32E-01
ctx-lh-rostralmiddlefrontal_26	476.4	466.0	1.02	2.92E-01
ctx-lh-superiortemporal_6	477.5	467.1	1.02	1.98E-01
ctx-rh-superiorparietal_20	584.3	571.5	1.02	1.92E-01
ctx-rh-precentral_29	693.2	677.9	1.02	1.72E-01
ctx-rh-insula_6	427.0	417.4	1.02	1.01E-01
ctx-rh-lingual_2	431.1	421.3	1.02	2.07E-01
ctx-lh-lingual_2	558.3	545.5	1.02	1.32E-01
ctx-lh-fusiform_11	348.7	340.7	1.02	2.17E-01
ctx-lh-supramarginal_6	563.6	550.7	1.02	1.59E-01
ctx-lh-transversetemporal_2	517.5	505.5	1.02	3.75E-02
ctx-lh-precentral_13	630.5	615.8	1.02	1.41E-01
ctx-lh-lateraloccipital_3	688.8	672.7	1.02	1.75E-01
ctx-lh-precentral_12	418.6	408.8	1.02	1.03E-01

ctx-lh-cuneus_5	633.7	618.7	1.02	1.03E-01
ctx-lh-lateraloccipital_15	767.6	749.3	1.02	1.34E-01
ctx-lh-caudalanteriorcingulate_5	828.6	808.7	1.02	3.57E-02
ctx-lh-insula_7	395.5	385.9	1.02	9.29E-02
ctx-rh-caudalmiddlefrontal_10	636.7	621.2	1.02	1.45E-01
ctx-lh-lingual_6	684.4	667.6	1.03	2.79E-01
ctx-lh-superiorparietal_12	603.4	588.6	1.03	1.67E-01
ctx-rh-paracentral_9	515.3	502.4	1.03	9.40E-02
ctx-rh-medialorbitofrontal_2	251.7	245.4	1.03	1.96E-01
ctx-rh-paracentral_6	481.7	469.4	1.03	7.83E-02
ctx-rh-pericalcarine_2	871.0	848.6	1.03	7.05E-02
Right-Medio_Dorsal	936.7	912.6	1.03	4.80E-02
ctx-lh-precentral_31	534.8	521.0	1.03	1.02E-01
ctx-rh-parsopercularis_9	451.9	440.1	1.03	1.32E-01
ctx-lh-precentral_9	542.0	527.9	1.03	1.39E-01
ctx-rh-frontalpole_2	349.7	340.6	1.03	1.96E-01
ctx-lh-caudalmiddlefrontal_3	834.2	812.4	1.03	6.94E-02
ctx-lh-posteriorcingulate_6	701.3	682.9	1.03	8.39E-02
ctx-lh-supramarginal_11	598.8	583.0	1.03	1.00E-01
ctx-lh-superiorparietal_29	246.3	239.6	1.03	1.18E-01
ctx-rh-superiorparietal_11	482.8	469.6	1.03	1.77E-01
ctx-rh-postcentral_9	592.6	576.3	1.03	1.40E-01
ctx-rh-precuneus_20	659.2	641.1	1.03	1.31E-01
ctx-rh-lingual_12	687.4	668.5	1.03	3.55E-02
ctx-lh-insula_15	381.4	370.8	1.03	1.07E-01
ctx-rh-postcentral_30	455.8	443.1	1.03	4.95E-02
ctx-lh-superiorfrontal_17	982.8	955.3	1.03	1.82E-02
ctx-lh-isthmuscingulate_4	646.9	628.8	1.03	9.80E-03
ctx-lh-supramarginal_5	480.2	466.6	1.03	4.09E-02
ctx-lh-lingual_9	724.9	704.3	1.03	3.39E-02
ctx-rh-superiorfrontal_42	510.7	496.1	1.03	1.31E-01
ctx-lh-cuneus_3	861.4	836.7	1.03	1.86E-02
ctx-rh-superiorfrontal_13	625.5	607.6	1.03	8.06E-02
ctx-lh-cuneus_1	557.4	541.2	1.03	2.36E-02
ctx-lh-postcentral_21	545.9	530.0	1.03	4.59E-02
ctx-rh-inferiorparietal_24	641.4	622.7	1.03	6.18E-02
ctx-rh-superiorfrontal_41	413.1	400.9	1.03	1.22E-01
ctx-rh-supramarginal_19	539.3	523.1	1.03	9.78E-02
ctx-rh-postcentral_22	478.0	463.7	1.03	3.87E-02
ctx-lh-lingual_1	652.6	633.0	1.03	1.30E-01
ctx-rh-precentral_17	462.4	448.5	1.03	5.16E-02
ctx-lh-superiorparietal_14	515.8	500.2	1.03	1.09E-01
ctx-lh-isthmuscingulate_5	619.5	600.4	1.03	3.51E-03
ctx-lh-posteriorcingulate_3	522.6	506.3	1.03	3.43E-02
ctx-lh-paracentral_2	363.0	351.4	1.03	5.14E-02
ctx-rh-supramarginal_6	543.3	525.7	1.03	6.13E-02
ctx-rh-postcentral_13	371.2	359.2	1.03	3.95E-02
ctx-rh-superiorfrontal_24	524.5	507.6	1.03	6.68E-02
ctx-lh-superiorparietal_11	401.7	388.6	1.03	9.21E-02
ctx-rh-precentral_20	615.3	595.2	1.03	2.71E-02

ctx-rh-precentral_12	392.8	380.0	1.03	4.34E-02
ctx-lh-superiortemporal_11	533.0	515.4	1.03	1.29E-02
ctx-lh-precuneus_23	625.6	605.0	1.03	4.13E-02
ctx-rh-superiorparietal_12	530.2	512.6	1.03	8.06E-02
ctx-lh-postcentral_13	401.1	387.8	1.03	3.18E-02
ctx-lh-precuneus_6	560.3	541.2	1.04	1.51E-02
ctx-lh-lateraloccipital_10	529.9	511.7	1.04	2.57E-02
ctx-rh-precentral_32	563.4	544.0	1.04	2.04E-02
ctx-lh-caudalmiddlefrontal_11	514.7	496.9	1.04	2.67E-02
ctx-lh-posteriorcingulate_8	438.4	423.1	1.04	1.02E-02
ctx-lh-paracentral_6	555.3	535.8	1.04	7.10E-03
ctx-rh-parsopercularis_2	577.7	557.4	1.04	3.25E-02
ctx-rh-pericalcarine_5	662.9	639.6	1.04	7.81E-02
ctx-lh-superiorparietal_10	913.8	881.6	1.04	4.63E-02
ctx-rh-precuneus_2	596.6	575.4	1.04	3.21E-02
ctx-lh-postcentral_8	607.7	586.0	1.04	2.49E-02
Left-Caudate	6991.8	6741.5	1.04	1.33E-03
ctx-rh-precentral_11	629.9	607.2	1.04	3.23E-02
ctx-rh-precuneus_16	707.9	682.3	1.04	2.12E-02
ctx-lh-paracentral_11	606.7	584.6	1.04	1.42E-02
ctx-lh-caudalmiddlefrontal_5	557.8	536.9	1.04	3.31E-02
ctx-rh-supramarginal_12	555.1	534.3	1.04	1.23E-02
ctx-lh-postcentral_5	628.5	604.9	1.04	7.02E-03
ctx-rh-paracentral_3	827.7	796.5	1.04	9.09E-03
ctx-rh-insula_1	524.2	504.5	1.04	1.95E-02
ctx-lh-pericalcarine_6	480.9	462.7	1.04	1.62E-02
ctx-rh-insula_11	555.6	534.5	1.04	9.59E-03
ctx-lh-paracentral_3	571.6	549.6	1.04	1.88E-02
ctx-lh-precentral_26	591.1	568.0	1.04	9.74E-03
ctx-lh-precuneus_20	652.1	626.5	1.04	5.24E-02
ctx-lh-caudalmiddlefrontal_8	929.4	892.6	1.04	5.96E-03
ctx-lh-precentral_19	469.8	451.2	1.04	1.40E-02
ctx-rh-precentral_7	707.5	679.4	1.04	3.22E-02
ctx-lh-superiorfrontal_42	470.8	452.1	1.04	3.22E-02
ctx-lh-paracentral_10	335.0	321.6	1.04	9.93E-03
ctx-rh-paracentral_2	738.9	708.9	1.04	2.19E-03
ctx-lh-precuneus_10	485.5	465.6	1.04	6.98E-03
ctx-rh-precentral_27	554.7	531.9	1.04	1.51E-02
ctx-lh-insula_6	525.2	503.5	1.04	7.37E-04
ctx-rh-postcentral_27	464.0	444.5	1.04	3.88E-03
ctx-lh-caudalmiddlefrontal_10	675.4	647.0	1.04	7.90E-03
ctx-rh-parstriangularis_8	557.5	534.0	1.04	1.55E-02
ctx-lh-precentral_32	560.0	536.4	1.04	3.09E-03
ctx-rh-transversetemporal_2	417.8	400.0	1.04	7.21E-04
ctx-rh-insula_16	425.0	406.9	1.04	2.46E-02
ctx-lh-paracentral_12	476.7	456.4	1.04	2.60E-03
ctx-rh-precentral_26	572.8	548.2	1.04	7.73E-03
ctx-rh-paracentral_7	550.5	526.4	1.05	2.16E-02
ctx-lh-lateralorbitofrontal_9	543.1	519.0	1.05	3.61E-03
ctx-rh-supramarginal_11	687.3	656.6	1.05	1.23E-02

ctx-rh-precentral_15	590.3	563.7	1.05	7.08E-03
ctx-lh-insula_11	756.6	722.4	1.05	3.12E-04
ctx-lh-postcentral_7	474.4	452.8	1.05	6.69E-04
ctx-lh-precentral_17	739.9	706.0	1.05	5.84E-04
ctx-rh-precentral_30	513.1	489.5	1.05	3.49E-03
ctx-lh-precuneus_16	563.5	537.5	1.05	1.08E-02
ctx-lh-superiorfrontal_31	1024.5	976.5	1.05	1.03E-03
ctx-lh-postcentral_27	558.1	531.9	1.05	7.91E-04
ctx-lh-pericalcarine_7	764.0	728.1	1.05	7.49E-04
ctx-rh-postcentral_21	718.4	684.5	1.05	3.15E-03
ctx-lh-transversetemporal_3	411.5	392.1	1.05	5.87E-04
ctx-lh-superiorfrontal_41	502.7	478.4	1.05	5.60E-03
ctx-lh-precentral_15	438.3	416.8	1.05	3.14E-03
ctx-lh-lateralorbitofrontal_6	1050.6	998.7	1.05	1.12E-03
ctx-lh-postcentral_30	515.5	489.9	1.05	3.51E-04
ctx-lh-superiorparietal_3	699.2	664.0	1.05	2.15E-03
ctx-lh-paracentral_9	580.2	550.9	1.05	5.84E-04
ctx-rh-cuneus_8	627.8	596.1	1.05	1.15E-04
ctx-lh-superiortemporal_14	662.8	629.2	1.05	2.71E-03
ctx-lh-insula_16	448.7	425.8	1.05	4.18E-03
ctx-rh-superiortemporal_11	616.3	584.8	1.05	4.95E-05
ctx-lh-precuneus_2	337.2	319.9	1.05	1.16E-02
ctx-rh-paracentral_4	587.5	557.4	1.05	5.54E-04
ctx-rh-insula_15	336.7	319.2	1.05	7.83E-03
ctx-lh-postcentral_29	575.7	545.6	1.06	1.76E-03
ctx-lh-insula_10	655.4	621.0	1.06	1.22E-03
ctx-rh-precentral_16	487.4	461.3	1.06	6.10E-04
ctx-rh-precentral_23	502.2	475.0	1.06	2.36E-03
ctx-rh-postcentral_5	515.3	487.3	1.06	1.40E-03
ctx-lh-precentral_7	453.3	428.3	1.06	3.82E-03
ctx-lh-precentral_11	605.2	571.6	1.06	1.17E-03
ctx-rh-precentral_24	540.1	509.9	1.06	5.63E-04
ctx-rh-precentral_22	505.6	477.2	1.06	6.04E-04
ctx-lh-parsopercularis_9	608.9	574.5	1.06	1.52E-04
Brain_Stem-SCP	162.6	153.4	1.06	3.48E-02
ctx-rh-transversetemporal_3	345.1	325.6	1.06	5.48E-05
ctx-rh-postcentral_29	531.6	501.5	1.06	1.82E-03
ctx-rh-pericalcarine_1	648.9	611.8	1.06	5.44E-03
ctx-rh-precuneus_3	560.0	527.9	1.06	6.72E-04
ctx-lh-precentral_27	578.4	545.1	1.06	8.72E-04
ctx-lh-medialorbitofrontal_11	552.2	520.4	1.06	4.99E-05
ctx-rh-postcentral_19	433.1	408.1	1.06	7.15E-05
ctx-lh-pericalcarine_2	655.1	616.7	1.06	3.04E-04
ctx-rh-postcentral_7	380.2	357.6	1.06	2.72E-04
ctx-lh-postcentral_14	512.2	481.5	1.06	5.76E-06
ctx-rh-precentral_33	565.0	531.0	1.06	4.36E-04
ctx-lh-parsopercularis_2	415.1	390.2	1.06	1.74E-04
ctx-lh-supramarginal_17	320.0	300.6	1.06	3.64E-04
ctx-lh-insula_3	408.4	383.6	1.06	2.06E-04
ctx-lh-paracentral_4	646.6	606.7	1.07	5.24E-06

ctx-lh-insula_13	726.8	681.4	1.07	6.42E-06
ctx-rh-supramarginal_17	685.2	642.0	1.07	3.67E-04
ctx-rh-postcentral_2	487.4	456.6	1.07	2.32E-06
ctx-rh-paracentral_10	503.8	471.9	1.07	9.68E-04
ctx-rh-paracentral_5	524.4	491.1	1.07	1.65E-04
ctx-rh-paracentral_8	471.6	441.4	1.07	8.56E-04
ctx-rh-precuneus_1	574.1	536.9	1.07	5.86E-05
ctx-rh-postcentral_14	419.3	391.9	1.07	1.11E-05
ctx-lh-precentral_33	592.1	552.7	1.07	3.40E-04
ctx-lh-pericalcarine_5	777.6	725.8	1.07	5.85E-04
ctx-lh-postcentral_19	376.5	351.2	1.07	2.31E-06
ctx-lh-postcentral_2	540.3	503.4	1.07	1.53E-08
ctx-lh-pericalcarine_1	950.0	884.5	1.07	5.61E-04
ctx-lh-paracentral_7	414.5	385.7	1.07	4.14E-04
ctx-lh-paracentral_8	384.3	357.4	1.08	4.44E-04
ctx-lh-insula_1	602.2	558.9	1.08	1.60E-05
ctx-rh-superiortemporal_14	447.8	415.2	1.08	2.48E-05
ctx-lh-cuneus_8	733.4	679.3	1.08	5.91E-08
ctx-lh-precentral_16	394.5	364.6	1.08	2.05E-06
ctx-lh-precentral_22	510.2	471.4	1.08	1.34E-05
ctx-lh-parstriangularis_8	615.6	567.6	1.08	2.69E-05
ctx-lh-precentral_23	452.8	417.4	1.08	1.18E-06
ctx-rh-insula_13	565.9	521.2	1.09	9.29E-07
ctx-lh-precentral_29	629.8	578.8	1.09	7.95E-07
ctx-lh-paracentral_5	526.7	483.3	1.09	1.81E-07
ctx-lh-precuneus_1	388.5	354.7	1.10	1.02E-05
ctx-lh-precuneus_3	372.3	339.2	1.10	9.37E-07
ctx-lh-precentral_24	559.0	508.6	1.10	1.51E-06
Brain_Stem-Medulla	798.0	724.2	1.10	1.98E-03
