# Supplementary material for: On the Increased and Decreased Structural Connectivity of the Demented Human Brain
Source: ArXiv. 2026 Jul 6:arXiv:2607.05654v1. Preprint. [Version 1] (PMC13370593)
Supplement: Supplement 1 [file NIHPP2607.05654v1-supplement-1.pdf]

Appendix

Node attributes

Here we describe the node- and edge attributes in the GraphML files published in

Node attributes

The node attributes in the GraphML files include the following values:

- dn_region can be cortical or subcortical
- dn_position_x, dn_position_y, dn_position_z the coordinates of a vertex. In a few cases, mostly in the substructures of the hippocampus, their values are missing if the substructure is not identified reliably. In that case the NAN abbreviation (not a number) is given there.
- dn_name the corresponding anatomical name; it is either identical to or a refinement of the fsname attribute.
- dn_hemisphere left or right
- dn_fsname the corresponding anatomical area name of the node in FreeSurfer.
- dn_multiscaleID numerical node ID

  When in lower resolutions the sub-partitioning of small areas, mostly subcortical structures, cannot be distinguished from each another (e.g., the finer partitioning of the hippocampus) then the coordinate-fields contain the word "nan".

Edge attributes

The edge attributes in the GraphML files include the following quantities:

- number_of_fibers, corresponding to an edge.
- fiber_length mean, median and standard deviation (std) of the fiber lengths in mm, corresponding to an edge.
- fiber_density, normalized_fiber_density, fiber_proportion: Since fibers (or streamlines) can not always be tracked reliably in tractography algorithms, and since fibers may start or end erroneously in white matter during tractography (which is possible only in gray matter anatomically), some authors prefer to use fiber density quantities instead or besides of fiber numbers [60].
- The following quantities are related to the image reconstruction method SHORE: Simple harmonic oscillator based reconstruction and estimation [61, 62, 63]:
- shore_rtop_signal: RTOP: Return-to-Origin Probability [64].
- shore_msd: MSD: Mean Squared Displacement, with standard deviation (std), mean and median values; [65, 66]
- shore_gfa: GFA: derived Generalized Fractional Anisotropy (GFA) with standard deviation (std), mean and median values [67];

Demented-healthy change for all vertices

The following table contains the averaged fiber-number-weighted vertex degree for healthy and demented subjects. In the computation, we have use the graphs of 351 demented and 624 healthy subjects, where "demented" include graphs, with corresponding subjects, which were not diagnosed as "demented" in the time of MRI recording, but in later sessions they were.

Name	Demented	Healthy	D/H Ratio
Left-Hippocampus_Fimbria	102.14	132.46	0.77
Left-Hippocampus_HATA	52.97	67.68	0.78
Right-Hippocampus_Fimbria	93.98	116.26	0.81
Right-Hippocampus_HATA	55.70	68.88	0.81
Right-Hippocampus_Parasubiculum	80.19	98.02	0.82
Left-Hippocampus	3 177.65	3 854.26	0.82
Right-Hippocampus	3 377.75	4 011.79	0.84
Left-Hippocampus_Tail	1 027.08	1 211.40	0.85
Right-Hippocampus_Tail	1 019.61	1 194.71	0.85
ctx-lh-fusiform_1	458.12	530.18	0.86
ctx-lh-parahippocampal_4	532.87	616.35	0.86
ctx-rh-parahippocampal_4	416.60	481.12	0.87
ctx-lh-entorhinal_2	615.69	710.44	0.87
ctx-rh-entorhinal_2	618.68	713.07	0.87
ctx-rh-entorhinal_1	724.12	832.11	0.87
ctx-rh-fusiform_1	558.20	641.22	0.87
ctx-lh-middletemporal_19	597.05	684.14	0.87
Left-Central_Lateral-Lateral_Posterior-Medial_Pulvinar	991.81	1 131.21	0.88
Left-Hippocampus_Parasubiculum	76.89	87.64	0.88
ctx-lh-middletemporal_9	616.06	698.98	0.88
ctx-lh-entorhinal_1	765.52	864.37	0.89
ctx-rh-middletemporal_19	528.76	595.94	0.89
ctx-lh-inferiortemporal_11	524.50	590.48	0.89
Right-Ventral_Latero_Dorsal	1 293.04	1 452.53	0.89
Left-Ventral_Latero_Dorsal	1 384.27	1 553.02	0.89
Left-Amygdala	1 482.89	1 659.08	0.89
ctx-lh-middletemporal_2	498.89	558.09	0.89
ctx-lh-inferiortemporal_1	474.52	529.05	0.90
ctx-rh-middletemporal_18	489.91	545.90	0.90
ctx-rh-middletemporal_5	342.28	381.29	0.90
ctx-rh-inferiortemporal_5	655.19	729.78	0.90
ctx-lh-temporalpole_2	695.39	774.38	0.90
ctx-rh-inferiortemporal_11	626.46	697.33	0.90
ctx-rh-superiortemporal_1	420.18	467.63	0.90
ctx-lh-middletemporal_18	515.45	571.34	0.90
ctx-rh-caudalmiddlefrontal_2	577.20	639.64	0.90
ctx-lh-parahippocampal_5	526.68	583.56	0.90
ctx-lh-middletemporal_1	396.22	438.93	0.90
ctx-lh-superiortemporal_1	454.47	501.41	0.91
ctx-lh-inferiortemporal_16	564.46	622.42	0.91
ctx-rh-parahippocampal_5	513.04	564.92	0.91
ctx-lh-inferiortemporal_5	683.23	750.07	0.91
ctx-rh-inferiortemporal_16	715.35	785.10	0.91

ctx-rh-middletemporal_9	679.52	745.74	0.91
ctx-rh-superiortemporal_10	319.31	350.12	0.91
ctx-rh-middletemporal_2	496.85	544.29	0.91
ctx-rh-superiorfrontal_29	461.62	505.11	0.91
ctx-rh-temporalpole_2	690.55	754.88	0.91
Right-Accumbens_area	1 380.89	1 508.92	0.92
ctx-lh-superiorfrontal_29	452.84	494.67	0.92
ctx-lh-middletemporal_7	361.59	394.42	0.92
ctx-lh-inferiortemporal_9	373.09	406.68	0.92
ctx-lh-fusiform_10	633.88	690.21	0.92
ctx-rh-fusiform_3	512.32	556.26	0.92
ctx-rh-superiorfrontal_7	621.18	674.09	0.92
ctx-rh-inferiortemporal_8	389.50	422.04	0.92
ctx-rh-caudalmiddlefrontal_9	925.70	1 002.31	0.92
ctx-lh-temporalpole_3	335.05	362.55	0.92
ctx-rh-temporalpole_3	319.79	345.70	0.93
ctx-rh-lateralorbitofrontal_4	556.07	600.18	0.93
Right-Amygdala	1 577.08	1 701.79	0.93
ctx-rh-inferiortemporal_12	510.32	550.60	0.93
Right-Central_Lateral-Lateral_Posterior-Medial_Pulvinar	868.32	936.16	0.93
ctx-lh-medialorbitofrontal_1	687.50	741.05	0.93
Left-Accumbens_area	1 358.12	1 463.33	0.93
ctx-lh-middletemporal_5	431.79	464.87	0.93
ctx-lh-middletemporal_6	412.64	443.88	0.93
ctx-rh-lateralorbitofrontal_7	581.02	624.32	0.93
ctx-lh-superiorfrontal_9	553.09	594.30	0.93
ctx-rh-fusiform_14	793.15	851.75	0.93
ctx-rh-medialorbitofrontal_10	741.46	795.90	0.93
ctx-rh-rostralmiddlefrontal_2	748.98	803.66	0.93
Left-Ventral_Latero_Ventral	1 855.79	1 990.18	0.93
ctx-rh-inferiortemporal_3	524.88	562.47	0.93
ctx-rh-inferiortemporal_7	741.26	793.46	0.93
ctx-lh-superiorfrontal_4	541.48	579.47	0.93
ctx-lh-lateralorbitofrontal_7	591.70	633.08	0.93
ctx-rh-lateralorbitofrontal_3	640.77	685.52	0.93
ctx-rh-superiorfrontal_20	596.69	638.28	0.93
ctx-rh-temporalpole_1	319.89	342.15	0.93
ctx-lh-lateralorbitofrontal_15	374.64	400.68	0.94
ctx-rh-caudalmiddlefrontal_4	542.13	579.70	0.94
ctx-lh-inferiortemporal_14	685.81	733.32	0.94
ctx-lh-lateralorbitofrontal_4	641.22	685.23	0.94
Right-Ventral_Latero_Ventral	1 992.75	2 129.48	0.94
ctx-rh-precentral_10	717.56	766.47	0.94
ctx-lh-fusiform_3	527.66	563.61	0.94
ctx-rh-bankssts_6	479.48	511.83	0.94
ctx-rh-middletemporal_11	387.79	413.86	0.94
ctx-rh-inferiortemporal_9	363.80	388.25	0.94
Brain_Stem-Pons	2 725.22	2 906.85	0.94
ctx-lh-inferiortemporal_2	756.41	806.76	0.94
ctx-lh-precuneus_14	644.24	686.93	0.94

Left-Pulvinar	1 363.62	1 453.85	0.94
ctx-lh-inferiortemporal_4	607.34	646.78	0.94
ctx-lh-superiorfrontal_10	486.35	517.86	0.94
ctx-lh-parstriangularis_4	470.26	500.69	0.94
ctx-rh-inferiortemporal_1	560.56	596.66	0.94
ctx-rh-superiortemporal_8	468.87	499.06	0.94
Right-Pulvinar	1 377.17	1 465.34	0.94
ctx-rh-fusiform_11	323.03	343.70	0.94
ctx-rh-fusiform_7	388.87	413.66	0.94
ctx-rh-precentral_4	616.62	655.65	0.94
ctx-lh-inferiortemporal_10	818.51	870.27	0.94
ctx-rh-rostralmiddlefrontal_1	624.46	663.73	0.94
ctx-rh-rostralmiddlefrontal_15	650.40	691.17	0.94
ctx-lh-lateralorbitofrontal_8	347.86	369.46	0.94
ctx-lh-middletemporal_11	352.23	374.04	0.94
ctx-lh-supramarginal_18	580.14	615.59	0.94
ctx-lh-caudalmiddlefrontal_2	710.45	753.77	0.94
ctx-rh-fusiform_10	665.43	705.92	0.94
ctx-rh-inferiortemporal_10	594.52	630.57	0.94
ctx-lh-middletemporal_3	554.78	587.95	0.94
ctx-lh-superiortemporal_20	702.75	744.63	0.94
ctx-rh-fusiform_6	383.11	405.88	0.94
ctx-lh-inferiortemporal_3	582.04	616.32	0.94
ctx-rh-medialorbitofrontal_3	699.61	740.35	0.94
ctx-rh-lateralorbitofrontal_14	708.43	749.62	0.95
ctx-rh-fusiform_13	760.45	804.64	0.95
ctx-lh-medialorbitofrontal_3	637.98	674.97	0.95
ctx-lh-rostralmiddlefrontal_1	580.37	613.85	0.95
ctx-lh-rostralmiddlefrontal_15	624.95	660.98	0.95
ctx-rh-superiorfrontal_1	603.83	638.56	0.95
ctx-rh-parstriangularis_4	624.91	660.61	0.95
ctx-lh-lateralorbitofrontal_3	710.15	750.19	0.95
ctx-rh-middletemporal_1	383.76	405.39	0.95
ctx-rh-fusiform_4	593.10	626.43	0.95
ctx-lh-lateralorbitofrontal_16	427.37	451.35	0.95
ctx-rh-medialorbitofrontal_1	706.87	746.43	0.95
ctx-lh-superiorfrontal_8	589.25	622.20	0.95
ctx-lh-inferiortemporal_12	536.49	566.46	0.95
Left-Hippocampus_Hippocampal_fissure	233.81	246.79	0.95
ctx-lh-parsorbitalis_3	436.19	460.37	0.95
ctx-rh-lateralorbitofrontal_13	509.00	537.15	0.95
ctx-rh-bankssts_4	545.08	575.20	0.95
ctx-rh-rostralmiddlefrontal_12	645.31	680.89	0.95
ctx-rh-superiortemporal_20	518.47	546.99	0.95
ctx-lh-medialorbitofrontal_9	638.10	673.14	0.95
ctx-lh-middletemporal_17	448.71	473.27	0.95
ctx-lh-medialorbitofrontal_5	427.10	450.25	0.95
ctx-rh-superiortemporal_4	276.25	291.21	0.95
ctx-rh-middletemporal_15	982.52	1 035.35	0.95
ctx-rh-parstriangularis_1	645.09	679.74	0.95

ctx-lh-superiorfrontal_36	636.60	670.75	0.95
ctx-rh-parsorbitalis_4	687.35	724.19	0.95
ctx-rh-parsopercularis_7	566.72	597.03	0.95
ctx-lh-caudalmiddlefrontal_9	1 092.18	1 150.52	0.95
ctx-lh-inferiortemporal_8	370.03	389.68	0.95
ctx-lh-lateraloccipital_7	649.31	683.77	0.95
ctx-lh-bankssts_4	647.15	681.49	0.95
ctx-rh-middletemporal_10	485.03	510.70	0.95
ctx-lh-precuneus_11	616.81	649.40	0.95
ctx-lh-superiorparietal_28	592.36	623.48	0.95
ctx-rh-inferiortemporal_2	596.65	627.96	0.95
ctx-lh-inferiorparietal_6	553.44	582.26	0.95
ctx-rh-superiortemporal_7	522.57	549.68	0.95
ctx-rh-middletemporal_7	393.42	413.81	0.95
ctx-lh-superiorparietal_16	540.94	568.95	0.95
ctx-lh-superiorfrontal_39	454.63	478.11	0.95
ctx-rh-superiorfrontal_36	618.13	650.06	0.95
ctx-lh-inferiorparietal_2	724.80	762.10	0.95
ctx-rh-parsorbitalis_1	481.06	505.78	0.95
ctx-rh-rostralmiddlefrontal_25	596.15	626.63	0.95
ctx-lh-middletemporal_8	388.30	408.14	0.95
ctx-rh-middletemporal_17	499.30	524.77	0.95
ctx-rh-caudalanteriorcingulate_1	749.59	787.28	0.95
ctx-lh-superiorfrontal_20	623.34	654.67	0.95
ctx-rh-parsopercularis_6	605.50	635.89	0.95
ctx-rh-lateralorbitofrontal_12	589.29	618.84	0.95
ctx-lh-parahippocampal_1	538.56	565.54	0.95
ctx-rh-superiorfrontal_32	607.13	637.45	0.95
ctx-rh-middletemporal_16	762.38	800.33	0.95
ctx-rh-parsorbitalis_3	632.94	664.21	0.95
ctx-lh-caudalanteriorcingulate_1	521.12	546.74	0.95
ctx-lh-middletemporal_13	435.59	456.99	0.95
ctx-lh-inferiortemporal_7	736.17	772.32	0.95
ctx-lh-superiortemporal_8	519.52	544.67	0.95
ctx-lh-middletemporal_15	739.21	774.75	0.95
ctx-rh-lateraloccipital_18	734.17	769.46	0.95
ctx-rh-superiorfrontal_4	662.21	693.99	0.95
ctx-rh-rostralmiddlefrontal_24	1 088.39	1 140.23	0.95
ctx-rh-parsorbitalis_2	616.67	645.96	0.95
ctx-rh-rostralmiddlefrontal_3	675.47	707.02	0.96
ctx-rh-superiorfrontal_10	505.70	529.29	0.96
ctx-lh-superiorparietal_21	348.84	365.05	0.96
ctx-rh-insula_8	372.26	389.54	0.96
ctx-rh-superiorparietal_28	605.56	633.66	0.96
ctx-lh-superiorfrontal_32	619.96	648.70	0.96
ctx-lh-rostralmiddlefrontal_2	799.96	836.53	0.96
ctx-rh-parahippocampal_6	279.75	292.50	0.96
ctx-lh-superiorfrontal_7	645.59	674.97	0.96
ctx-lh-parsorbitalis_1	564.17	589.84	0.96
ctx-lh-precentral_4	592.90	619.63	0.96

ctx-lh-supramarginal_15	761.29	795.46	0.96
ctx-lh-superiortemporal_10	376.45	393.29	0.96
ctx-lh-inferiorparietal_4	705.42	736.70	0.96
ctx-rh-superiorparietal_21	381.13	397.93	0.96
ctx-rh-superiorfrontal_23	613.98	641.03	0.96
ctx-lh-superiortemporal_18	654.96	683.60	0.96
ctx-rh-inferiortemporal_14	647.12	675.40	0.96
ctx-rh-parsopercularis_8	610.44	636.72	0.96
ctx-rh-superiorfrontal_30	590.81	616.18	0.96
ctx-lh-rostralmiddlefrontal_16	707.24	737.49	0.96
Left-Ventral_Anterior	1 398.18	1 457.93	0.96
ctx-rh-medialorbitofrontal_8	575.69	600.25	0.96
ctx-lh-rostralmiddlefrontal_14	735.53	766.89	0.96
ctx-rh-lateraloccipital_17	640.71	667.92	0.96
ctx-rh-precentral_3	614.21	640.27	0.96
ctx-rh-rostralmiddlefrontal_5	570.68	594.83	0.96
ctx-rh-parahippocampal_1	481.72	502.03	0.96
ctx-rh-inferiortemporal_15	852.60	888.49	0.96
ctx-lh-precentral_2	599.16	624.32	0.96
ctx-rh-inferiortemporal_4	522.34	544.25	0.96
ctx-rh-medialorbitofrontal_9	562.65	586.25	0.96
ctx-rh-rostralmiddlefrontal_17	596.70	621.72	0.96
ctx-lh-rostralanteriorcingulate_4	884.95	921.65	0.96
ctx-rh-superiortemporal_19	544.10	566.65	0.96
ctx-rh-lateraloccipital_22	601.34	626.23	0.96
ctx-rh-rostralmiddlefrontal_16	690.78	719.26	0.96
ctx-rh-middletemporal_13	518.87	540.26	0.96
ctx-rh-rostralmiddlefrontal_19	797.58	830.37	0.96
Right-Ventral_Anterior	1 354.87	1 410.55	0.96
ctx-rh-lateralorbitofrontal_10	463.05	482.06	0.96
ctx-lh-rostralmiddlefrontal_25	617.42	642.57	0.96
ctx-rh-medialorbitofrontal_4	406.62	423.12	0.96
ctx-lh-superiorparietal_17	553.20	575.62	0.96
ctx-lh-insula_2	420.62	437.63	0.96
ctx-lh-inferiorparietal_5	466.60	485.45	0.96
ctx-rh-superiorfrontal_8	595.93	619.72	0.96
ctx-rh-lateraloccipital_6	641.78	667.34	0.96
ctx-lh-lateralorbitofrontal_13	580.21	603.18	0.96
ctx-lh-middletemporal_10	409.49	425.64	0.96
ctx-lh-rostralmiddlefrontal_5	642.83	668.15	0.96
ctx-rh-supramarginal_15	602.74	626.45	0.96
ctx-rh-medialorbitofrontal_7	485.59	504.54	0.96
ctx-lh-inferiorparietal_19	550.36	571.81	0.96
ctx-rh-medialorbitofrontal_5	389.47	404.50	0.96
ctx-rh-superiorfrontal_9	610.41	633.77	0.96
ctx-lh-superiorparietal_2	423.54	439.59	0.96
ctx-rh-caudalmiddlefrontal_7	636.52	660.62	0.96
ctx-lh-rostralmiddlefrontal_27	500.43	519.32	0.96
ctx-lh-rostralmiddlefrontal_19	730.05	757.54	0.96
ctx-lh-inferiorparietal_7	480.09	498.13	0.96

ctx-rh-superiorparietal_25	594.41	616.72	0.96
ctx-rh-rostralanteriorcingulate_4	587.91	609.92	0.96
ctx-lh-precentral_6	625.87	649.26	0.96
ctx-rh-parstriangularis_2	722.11	748.93	0.96
ctx-lh-superiorfrontal_15	556.53	577.18	0.96
ctx-rh-parstriangularis_6	546.43	566.66	0.96
ctx-rh-rostralmiddlefrontal_13	590.74	612.56	0.96
ctx-rh-bankssts_5	732.71	759.76	0.96
ctx-rh-bankssts_3	539.68	559.58	0.96
ctx-rh-middletemporal_8	747.00	774.48	0.96
ctx-lh-medialorbitofrontal_8	600.09	622.12	0.96
ctx-lh-rostralmiddlefrontal_3	693.15	718.43	0.96
ctx-lh-rostralmiddlefrontal_11	455.75	472.26	0.97
ctx-lh-lateralorbitofrontal_14	834.99	865.18	0.97
ctx-lh-frontalpole_2	120.88	125.23	0.97
ctx-rh-parstriangularis_5	671.62	695.76	0.97
ctx-lh-lateralorbitofrontal_2	694.21	719.00	0.97
ctx-lh-precentral_10	860.74	891.29	0.97
ctx-lh-parsopercularis_3	522.26	540.79	0.97
ctx-rh-superiorfrontal_15	563.11	583.08	0.97
ctx-rh-superiorfrontal_12	580.47	601.00	0.97
ctx-lh-middletemporal_16	647.92	670.80	0.97
ctx-rh-superiorfrontal_39	460.69	476.80	0.97
ctx-lh-superiorparietal_25	641.13	663.50	0.97
ctx-rh-precentral_18	795.40	823.13	0.97
ctx-lh-insula_12	636.73	658.82	0.97
ctx-rh-lateraloccipital_16	758.84	785.05	0.97
ctx-rh-precentral_31	509.53	527.09	0.97
ctx-lh-superiorfrontal_1	631.89	653.66	0.97
ctx-lh-superiorfrontal_21	580.72	600.37	0.97
ctx-rh-fusiform_5	425.75	440.13	0.97
ctx-lh-precuneus_15	697.26	720.70	0.97
ctx-rh-precuneus_13	659.34	681.45	0.97
ctx-lh-superiorfrontal_37	578.08	597.44	0.97
ctx-lh-caudalmiddlefrontal_4	622.23	642.98	0.97
ctx-rh-parsopercularis_1	625.34	646.18	0.97
ctx-lh-postcentral_24	199.19	205.79	0.97
ctx-rh-precuneus_14	689.92	712.77	0.97
ctx-rh-rostralanteriorcingulate_2	955.40	986.97	0.97
ctx-rh-inferiorparietal_16	497.37	513.62	0.97
ctx-rh-superiorfrontal_33	638.52	659.36	0.97
ctx-rh-postcentral_25	484.73	500.52	0.97
ctx-lh-inferiorparietal_17	563.17	581.52	0.97
ctx-lh-precuneus_13	676.31	698.22	0.97
ctx-lh-fusiform_4	601.25	620.70	0.97
ctx-lh-parsopercularis_8	933.86	963.92	0.97
ctx-rh-rostralmiddlefrontal_6	778.63	803.68	0.97
ctx-lh-superiortemporal_19	480.49	495.92	0.97
ctx-rh-precentral_6	594.09	613.14	0.97
ctx-rh-caudalanteriorcingulate_2	547.64	565.19	0.97

ctx-rh-fusiform_8	581.28	599.88	0.97
ctx-lh-superiorfrontal_30	615.42	635.05	0.97
ctx-lh-precuneus_18	642.66	663.11	0.97
ctx-rh-lateraloccipital_11	795.38	820.66	0.97
ctx-rh-inferiorparietal_15	618.97	638.49	0.97
ctx-rh-rostralmiddlefrontal_8	445.54	459.57	0.97
ctx-lh-rostralanteriorcingulate_2	1 226.47	1 264.90	0.97
ctx-lh-rostralmiddlefrontal_9	969.87	1 000.06	0.97
ctx-lh-medialorbitofrontal_7	573.01	590.83	0.97
ctx-rh-lateralorbitofrontal_1	600.54	619.20	0.97
ctx-lh-superiorparietal_27	640.61	660.46	0.97
ctx-lh-parahippocampal_6	275.83	284.33	0.97
ctx-rh-cuneus_3	694.45	715.71	0.97
ctx-rh-caudalanteriorcingulate_4	527.22	543.35	0.97
ctx-lh-precentral_18	871.60	898.12	0.97
ctx-rh-precentral_36	498.12	513.24	0.97
ctx-rh-supramarginal_20	607.11	625.50	0.97
ctx-lh-fusiform_13	623.79	642.65	0.97
ctx-lh-parsopercularis_4	785.67	809.40	0.97
ctx-lh-parsorbitalis_4	562.44	579.32	0.97
ctx-lh-superiortemporal_15	638.47	657.36	0.97
ctx-rh-superiortemporal_3	217.65	224.08	0.97
ctx-lh-superiorparietal_18	704.36	725.15	0.97
ctx-rh-precentral_2	613.89	631.92	0.97
ctx-lh-superiorfrontal_40	562.17	578.53	0.97
ctx-rh-inferiortemporal_13	1 089.76	1 121.46	0.97
ctx-rh-cuneus_7	679.26	698.96	0.97
ctx-rh-superiorfrontal_3	666.93	686.09	0.97
ctx-lh-middletemporal_12	702.74	722.79	0.97
ctx-lh-parsopercularis_6	585.32	602.01	0.97
ctx-lh-parstriangularis_6	233.43	240.07	0.97
ctx-rh-rostralmiddlefrontal_4	566.94	582.91	0.97
ctx-rh-superiorparietal_27	629.67	647.39	0.97
ctx-rh-superiorparietal_8	580.12	596.40	0.97
ctx-rh-superiorfrontal_35	667.76	686.45	0.97
ctx-lh-bankssts_6	511.24	525.54	0.97
ctx-rh-inferiorparietal_5	495.30	509.07	0.97
ctx-lh-superiorparietal_20	530.89	545.59	0.97
ctx-rh-parsopercularis_3	448.40	460.77	0.97
ctx-rh-rostralmiddlefrontal_11	569.44	585.12	0.97
ctx-lh-rostralmiddlefrontal_13	469.38	482.31	0.97
ctx-rh-parsopercularis_4	547.50	562.55	0.97
ctx-lh-superiorfrontal_5	703.69	722.93	0.97
ctx-rh-lateralorbitofrontal_17	468.25	481.03	0.97
ctx-lh-superiortemporal_12	538.58	553.28	0.97
ctx-lh-superiorfrontal_12	618.68	635.53	0.97
ctx-lh-superiorparietal_9	896.59	920.60	0.97
ctx-rh-lingual_8	672.48	690.46	0.97
ctx-rh-supramarginal_9	720.85	740.07	0.97
ctx-lh-precentral_3	603.85	619.90	0.97

ctx-lh-lateraloccipital_17	653.96	671.22	0.97
ctx-rh-superiorfrontal_21	540.15	554.28	0.97
ctx-rh-posteriorcingulate_9	438.56	449.98	0.97
ctx-rh-superiorfrontal_2	671.87	689.23	0.97
ctx-lh-inferiortemporal_15	906.95	930.33	0.97
ctx-rh-superiorparietal_24	593.99	609.26	0.97
ctx-lh-rostralmiddlefrontal_17	597.02	612.30	0.98
ctx-rh-cuneus_2	382.51	392.27	0.98
ctx-rh-middletemporal_12	967.03	991.68	0.98
ctx-rh-caudalanteriorcingulate_5	652.48	668.73	0.98
ctx-rh-superiorfrontal_18	659.55	675.90	0.98
ctx-rh-rostralmiddlefrontal_14	797.99	817.54	0.98
ctx-lh-rostralmiddlefrontal_8	434.60	445.22	0.98
ctx-lh-inferiorparietal_11	578.27	592.37	0.98
ctx-rh-superiortemporal_12	612.50	627.27	0.98
ctx-rh-superiorparietal_2	466.23	477.45	0.98
ctx-lh-supramarginal_20	671.05	687.07	0.98
ctx-lh-fusiform_8	521.47	533.88	0.98
ctx-rh-superiorparietal_17	471.59	482.78	0.98
ctx-lh-rostralmiddlefrontal_12	695.11	711.56	0.98
ctx-rh-inferiorparietal_10	682.29	698.40	0.98
ctx-lh-parstriangularis_5	771.20	789.39	0.98
ctx-rh-superiortemporal_16	572.22	585.67	0.98
ctx-lh-lateralorbitofrontal_12	650.15	665.42	0.98
ctx-lh-fusiform_16	495.04	506.65	0.98
ctx-lh-rostralmiddlefrontal_6	709.43	726.05	0.98
ctx-lh-insula_14	438.64	448.90	0.98
ctx-rh-rostralanteriorcingulate_3	630.99	645.67	0.98
ctx-lh-superiorfrontal_38	552.50	565.30	0.98
ctx-rh-medialorbitofrontal_11	623.47	637.86	0.98
ctx-rh-supramarginal_14	628.23	642.71	0.98
ctx-rh-inferiortemporal_6	792.73	810.93	0.98
ctx-lh-superiortemporal_2	446.82	457.06	0.98
ctx-lh-supramarginal_1	815.04	833.64	0.98
ctx-rh-fusiform_16	368.78	377.20	0.98
ctx-lh-inferiortemporal_13	994.34	1 016.95	0.98
ctx-rh-inferiorparietal_18	912.94	933.68	0.98
ctx-lh-lateraloccipital_23	681.16	696.64	0.98
ctx-rh-rostralmiddlefrontal_7	646.70	661.28	0.98
ctx-rh-posteriorcingulate_5	553.32	565.78	0.98
ctx-rh-middletemporal_3	512.05	523.53	0.98
ctx-rh-superiorfrontal_38	562.62	575.20	0.98
ctx-rh-lateralorbitofrontal_5	633.99	648.16	0.98
Right-Anterior	1 629.61	1 665.97	0.98
ctx-rh-insula_12	628.54	642.41	0.98
ctx-lh-temporalpole_1	545.56	557.46	0.98
Left-Anterior	1 709.09	1 746.29	0.98
ctx-rh-rostralmiddlefrontal_9	775.18	792.02	0.98
ctx-rh-lateraloccipital_2	717.08	732.62	0.98
ctx-rh-superiorfrontal_34	585.44	598.05	0.98

ctx-rh-parsopercularis_5	413.26	422.11	0.98
ctx-rh-supramarginal_3	621.75	635.02	0.98
ctx-lh-caudalanteriorcingulate_4	472.58	482.58	0.98
ctx-rh-precentral_1	598.63	611.20	0.98
ctx-rh-bankssts_2	656.90	670.64	0.98
ctx-lh-inferiorparietal_21	594.73	607.17	0.98
ctx-lh-superiorfrontal_23	721.84	736.72	0.98
ctx-rh-superiorparietal_5	639.03	652.18	0.98
ctx-rh-superiortemporal_18	610.99	623.39	0.98
ctx-rh-inferiorparietal_17	683.84	697.72	0.98
ctx-rh-precuneus_15	690.43	704.42	0.98
ctx-rh-postcentral_3	538.92	549.79	0.98
ctx-lh-supramarginal_4	503.09	513.24	0.98
ctx-lh-superiorfrontal_34	642.93	655.78	0.98
ctx-rh-fusiform_9	978.94	998.41	0.98
ctx-lh-superiortemporal_7	491.03	500.78	0.98
ctx-lh-parstriangularis_1	655.56	668.58	0.98
ctx-rh-superiortemporal_9	538.48	549.13	0.98
ctx-lh-caudalmiddlefrontal_7	728.07	742.46	0.98
ctx-lh-lateralorbitofrontal_1	494.61	504.27	0.98
ctx-rh-superiorparietal_16	542.50	553.05	0.98
ctx-lh-lateraloccipital_19	679.10	692.29	0.98
ctx-rh-superiorfrontal_14	595.35	606.88	0.98
ctx-lh-superiortemporal_3	245.25	249.96	0.98
ctx-rh-rostralmiddlefrontal_22	784.19	798.72	0.98
ctx-lh-supramarginal_3	742.73	756.42	0.98
ctx-rh-insula_2	502.37	511.60	0.98
ctx-rh-rostralmiddlefrontal_10	839.05	854.40	0.98
ctx-rh-superiortemporal_17	403.93	411.24	0.98
ctx-rh-supramarginal_8	807.32	821.89	0.98
ctx-lh-precuneus_7	783.72	797.84	0.98
ctx-rh-postcentral_18	445.72	453.75	0.98
ctx-lh-medialorbitofrontal_10	776.87	790.78	0.98
ctx-rh-cuneus_4	724.41	737.36	0.98
ctx-rh-rostralanteriorcingulate_1	620.49	631.54	0.98
ctx-rh-parstriangularis_3	495.37	504.10	0.98
ctx-lh-inferiorparietal_1	555.66	565.38	0.98
ctx-lh-supramarginal_10	749.13	762.21	0.98
ctx-lh-superiorparietal_13	976.24	993.27	0.98
ctx-lh-inferiorparietal_3	551.40	560.97	0.98
ctx-rh-lateraloccipital_19	701.71	713.84	0.98
ctx-lh-superiortemporal_17	416.92	424.10	0.98
ctx-lh-rostralmiddlefrontal_20	544.49	553.87	0.98
ctx-lh-posteriorcingulate_4	586.95	596.94	0.98
ctx-rh-superiorparietal_18	728.44	740.83	0.98
ctx-rh-superiorfrontal_40	566.55	576.12	0.98
ctx-lh-bankssts_1	613.60	623.89	0.98
ctx-rh-lingual_16	665.06	676.21	0.98
ctx-rh-precuneus_17	513.83	522.38	0.98
ctx-lh-caudalanteriorcingulate_3	616.36	626.46	0.98

ctx-rh-postcentral_6	634.99	645.39	0.98
ctx-rh-superiorfrontal_5	727.98	739.87	0.98
ctx-rh-fusiform_2	951.24	966.60	0.98
ctx-lh-middletemporal_14	544.08	552.79	0.98
ctx-rh-lateraloccipital_7	656.36	666.86	0.98
ctx-lh-superiorparietal_5	579.98	589.21	0.98
ctx-rh-inferiorparietal_21	703.28	714.43	0.98
ctx-lh-inferiorparietal_24	684.86	695.71	0.98
ctx-rh-precentral_21	285.01	289.50	0.98
ctx-rh-parstriangularis_7	634.53	644.50	0.98
ctx-rh-precentral_35	413.80	420.27	0.98
ctx-rh-lateraloccipital_1	611.11	620.63	0.98
ctx-rh-precentral_28	462.22	469.39	0.98
ctx-lh-precuneus_5	443.60	450.47	0.98
ctx-rh-precuneus_18	661.66	671.84	0.98
ctx-lh-inferiorparietal_25	871.25	884.64	0.98
ctx-lh-superiortemporal_23	478.70	486.02	0.98
Left-VentralDC	2 718.16	2 759.48	0.99
ctx-rh-lateralorbitofrontal_2	469.27	476.40	0.99
Left-Medio_Dorsal	1 071.73	1 087.98	0.99
ctx-rh-caudalanteriorcingulate_6	428.87	435.36	0.99
ctx-lh-lingual_11	604.93	614.06	0.99
ctx-rh-postcentral_20	495.70	503.08	0.99
ctx-lh-fusiform_15	496.12	503.44	0.99
ctx-lh-bankssts_2	669.24	679.10	0.99
ctx-rh-precuneus_11	617.91	627.00	0.99
ctx-rh-rostralmiddlefrontal_23	636.91	646.25	0.99
ctx-rh-cuneus_6	754.40	765.43	0.99
ctx-rh-rostralmiddlefrontal_27	531.12	538.87	0.99
ctx-lh-postcentral_25	605.70	614.52	0.99
ctx-rh-postcentral_15	389.18	394.84	0.99
ctx-rh-lateraloccipital_4	663.80	673.35	0.99
ctx-lh-superiorparietal_26	503.05	510.23	0.99
ctx-rh-inferiorparietal_13	859.94	872.06	0.99
ctx-rh-inferiorparietal_12	779.71	790.61	0.99
ctx-lh-bankssts_3	652.67	661.79	0.99
ctx-lh-medialorbitofrontal_4	467.62	474.07	0.99
ctx-rh-lateraloccipital_3	640.15	648.97	0.99
ctx-rh-insula_9	783.04	793.79	0.99
ctx-lh-precentral_1	611.50	619.86	0.99
ctx-lh-inferiorparietal_10	494.37	501.13	0.99
ctx-rh-postcentral_23	381.64	386.85	0.99
ctx-lh-inferiorparietal_16	425.02	430.77	0.99
ctx-rh-bankssts_1	768.81	779.20	0.99
ctx-rh-parahippocampal_2	383.74	388.92	0.99
ctx-rh-supramarginal_4	423.78	429.38	0.99
ctx-rh-cuneus_5	715.09	724.47	0.99
ctx-lh-precentral_35	422.83	428.38	0.99
ctx-lh-lateralorbitofrontal_5	719.36	728.75	0.99
ctx-lh-lateraloccipital_2	845.44	856.40	0.99
